# Lymphatic filariasis risk prediction models: A systematic review of modelling approaches, predictors, validation practices, and methodological quality

**DOI:** 10.1371/journal.pntd.0014843

**Published:** 2026-11-10

**Authors:** Hari Kishan Raju Konuganti, Vignesh Kumar Ramakrishnan, Niroshini Mani, Swetha Elumalai, Ashok Kumar Elango, Chakravarthi Madda, Indra Sara Lama, Manju Rahi

**Affiliations:** Division of Climate Change, GIS and VBD Stratification/Mapping ICMR National Institute for Vector Control Research, Department of Health Research, Ministry of Health & Family Welfare, GOI, Medical Complex, Indira Nagar, Puducherry, India; Institute of Continuing Medical Education of Ioannina, GREECE

## Abstract

**Background:**

Risk prediction models are increasingly used to support Lymphatic filariasis (LF) surveillance, identification of residual transmission foci, and elimination planning. However, their methodological quality, validation practices, and translational capacity have not been systematically evaluated.

**Methodology/principal findings:**

To address this gap, we conducted a systematic review following the Preferred Reporting Items for Systematic Reviews and Meta-Analyses (PRISMA) guidelines to characterise the methodological approaches, predictor domains, validation practices, model performance assessment, and risk of bias of published LF risk prediction models. The review was not prospectively registered. Studies developing statistical, Bayesian spatial, ecological niche, machine learning (ML), or hybrid models to estimate LF prevalence, infection status, or transmission risk were included. Data were extracted using the Checklist for Critical Appraisal and Data Extraction for Systematic Reviews of Prediction Modelling Studies (CHARMS), and risk of bias was assessed using the Prediction model Risk Of Bias ASsessment Tool (PROBAST). Twenty-six studies published between 2000 and 2025 were included. Most studies were conducted in Africa and India and relied on spatially aggregated epidemiological and environmental data. Climatic (73.1%) and environmental (76.9%) predictors predominated and frequently co-occurred, whereas socio-demographic (57.7%), programmatic (7.7%), and vector-related (7.7%) variables were less consistently incorporated. Bayesian and spatial models were mostly aligned with expert-driven predictor selection and internal validation, whereas ML and hybrid modelling approaches aligned with algorithm-based predictor selection and cross-validation. Despite increasing methodological diversification, external validation (15.4%) and calibration assessment (7.7%) were rarely reported. All studies were assessed as having high or unclear risk of bias in the analysis domain.

**Conclusions/significance:**

LF risk prediction modelling is methodologically diverse but remains predominantly reliant on internal validation. Limited external validation and calibration assessment constrain generalisability and reliability of published models. Improving external validation, calibration assessment, methodological transparency, and reporting standards will be essential for developing robust prediction models capable of supporting elimination verification and post-elimination surveillance.

## Author summary

Lymphatic filariasis (LF) is a mosquito-borne neglected tropical disease (NTD) that remains a public health challenge in many tropical and subtropical countries. As countries move towards eliminating the disease, prediction models are increasingly being used to identify areas where transmission may persist and to help guide surveillance and control activities. Although many prediction models have been published, their methodological quality has not previously been evaluated in a systematic way. In this review, we examined published LF risk prediction models using internationally recognised frameworks for assessing prediction model quality and risk of bias. We found that most studies relied heavily on climatic and environmental information, while fewer incorporated demographic, programme, or vector-related data. We also found that many models were evaluated only using the data on which they were developed, with relatively few being tested in independent settings. Important aspects of model evaluation, including calibration and transparent reporting, were also frequently lacking. Our findings highlight several opportunities to improve future LF prediction models. More rigorous validation, better reporting, and greater integration of epidemiological, environmental, and programme data will help produce prediction models that are more reliable and useful for supporting disease surveillance, elimination verification, and post-elimination monitoring.

## 1. Introduction

Lymphatic filariasis (LF) is a vector-borne neglected tropical disease (NTD) primarily caused by the filarial parasites *Wuchereria bancrofti*, *Brugia malayi*, and *Brugia timori*. It remains a significant cause of chronic morbidity, disability, social stigma, and economic loss in endemic populations [1,2]. Although the Global Programme to Eliminate Lymphatic Filariasis (GPELF) has made considerable progress, LF transmission still persists in several regions of Africa, Southeast Asia, especially India, and certain Pacific nations. In these regions, residual transmission foci and spatial heterogeneity continue to pose major challenges to elimination and post-elimination surveillance [3].

LF transmission results from complex interactions among humans, parasites, vectors, and a wide range of environmental and socio-ecological conditions. Climatic variables such as temperature, rainfall, and humidity influence vector survival and parasite development, while land-use land-cover (LULC) patterns, population density, sanitation, housing conditions, and socio-economic factors further shape LF transmission patterns [4,5]. These interactions generate substantial geographic variability in LF risk, often at very fine spatial scales, even within areas that have undergone prolonged transmission control activities. Consequently, uniform disease surveillance and control strategies may be insufficient to detect localized high-risk settings, highlighting the importance of predictive risk assessment.

Therefore, predictive modelling has become an important tool for understanding and estimating LF risk across diverse real-world settings. In contrast to intervention-focused or transmission dynamics models, which primarily simulate the impact of control strategies such as mass drug administration (MDA), risk prediction models aim to estimate the probability or intensity of LF occurrence based on associations between observed outcomes and explanatory predictors [4,6]. Such models are particularly relevant for identifying areas at high risk, guiding surveillance prioritisation, and supporting decision making in elimination and post-elimination strategies [3,7].

A wide spectrum of modelling approaches has been applied to LF risk prediction. These include conventional statistical models, geospatial and Bayesian hierarchical frameworks, Ecological Niche Modelling (ENM) and more recently, Artificial Intelligence (AI) and Machine Learning (ML) based models that are capable of handling nonlinear relationships in high dimensional data [4,7,8]. Across these modelling approaches, studies differ substantially in their choice of predictors such as climatic, environmental, demographic, socio-economic, and vector-related variables, as well as in the predictor selection strategies ranging from expert-driven inclusion and univariate screening to multivariable and algorithm-based screening methods [4,5]. Similarly, LF risk has been operationalised using diverse outcome definitions, such as infection prevalence, antigen or microfilaria positivity, or composite transmission risk indices [6,7].

Although general methodological guidance exists for prediction modelling, including recommendations for transparent reporting and assessment of risk of bias, LF specific risk prediction studies have not yet been systematically synthesised from a methodological perspective [9,10]. In particular, there has been no comprehensive review focusing exclusively on LF risk prediction models that examines how predictors are selected, how models are developed and validated, and how performance is assessed, while explicitly excluding intervention based modelling studies.

Despite the availability of these methodological frameworks, model evaluation practices remain highly heterogeneous across published LF risk prediction studies. Performance has been assessed using different combinations of discrimination, accuracy, and calibration metrics, while validation strategies range from internal approaches such as cross-validation or data splitting to more limited use of external or real-world validation [9,10]. Such heterogeneity in modelling choices, predictor handling, and validation practices complicates comparison across studies, limits the interpretability and generalisability of published models [9].

Therefore, this systematic review aims to systematically characterise published LF risk prediction models by examining their modelling approaches, predictor domains, validation practices, performance assessment, and methodological quality using established prediction modelling appraisal frameworks. By providing a structured methodological synthesis of published LF risk prediction models, this review seeks to identify current methodological strengths and limitations and to inform the development of more robust, transparent, and policy-relevant prediction models for elimination and post-elimination surveillance [9,10]. The main methodological and translational learning points identified from the review are summarised in Box 1.

**Box 1. Key learning points**

**Table:** 

LF risk prediction models show strong methodological clustering
Climatic and environmental predictors dominate modelling approaches
External validation and calibration assessment were rarely performed
Outcome definition and performance reporting heterogeneity limits comparability across studies
Most studies have high or unclear risk of bias in the analysis domain

## 2 Materials and methods

### 2.1 Study design

This systematic review aims to identify, synthesize, and critically evaluate studies focused on LF risk prediction modelling. This review was conducted following the Preferred Reporting Items for Systematic Reviews and Meta-Analyses (PRISMA) guidelines [11,12]. This review was not prospectively registered. Data extraction was guided by the Checklist for Critical Appraisal and Data Extraction for Systematic Reviews of Prediction Modelling Studies (CHARMS) framework, an established standard for systematic reviews of prediction models [13], and risk of bias was assessed using the Prediction model Risk Of Bias ASsessment Tool (PROBAST) [10].

### 2.2 Search strategy

A comprehensive and systematic literature search was carried out across multiple available databases such as Scopus, Web of Science, PubMed, and Google Scholar, to identify studies relevant to LF risk prediction modelling. These databases were chosen to ensure broader coverage of epidemiological, environmental, biomedical, and interdisciplinary modelling research. The final literature search was conducted on 9 January 2026.

Search strategies integrated controlled vocabulary with free-text terms relevant to LF and predictive modelling. Disease specific terms included “lymphatic filariasis”, “filariasis”, “Wuchereria bancrofti”, “Brugia malayi”, and “Brugia timori”, while modelling related terms encompassed a range of predictive approaches, such as “risk prediction”, “risk model”, “spatial model”, “geo-statistical model”, “time series”, “forecasting”, “ecological niche model”, “MaxEnt”, “machine learning”, “deep learning”, “artificial intelligence”, and “Bayesian”. Boolean operators such as AND/OR were used in combination with disease and modelling concepts, and truncation was applied where appropriate to capture relevant term variants.

Database-specific adaptations of the search strategy were implemented to address differences in indexing and search functionality. Searches were conducted within the title and abstract fields in PubMed, and within TITLE-ABS-KEY fields in Scopus, while topic searches (TS) were used in Web of Science. Google Scholar was searched using simplified keyword combinations to identify potentially relevant studies not indexed in traditional bibliographic databases. The complete search strings, including Boolean logic and field restrictions for each database, are provided in the S1 Material.

No restrictions were applied with respect to published year, geographic location or modelling approach. The literature search was restricted to peer-reviewed publications. All retrieved search records were exported for deduplication and subsequent screening.

Titles and abstracts were screened independently by three reviewers. Full-text articles deemed potentially eligible were assessed independently by the same three reviewers against the predefined inclusion and exclusion criteria. Discrepancies were resolved through group discussion among the reviewers followed by consensus. The earliest eligible study identified was published in 2000; no eligible LF risk prediction modelling studies published before 2000 were identified despite the unrestricted search period.

### 2.3 Eligibility criteria

#### 2.3.1 Inclusion criteria.

Studies were considered eligible if they developed, updated, validated, or externally evaluated statistical, geospatial, ML, ecological niche, or Bayesian prediction models for LF. Eligible studies aimed to predict the probability, prevalence, intensity, occurrence, environmental suitability, or spatial distribution of LF infection or transmission risk using observed epidemiological data in combination with demographic, environmental, climatic, socioeconomic, entomological, or spatial predictors. Only original peer-reviewed research articles published in English were included.

#### 2.3.2 Exclusion criteria.

We have excluded the studies if they:

- Primarily evaluated the impact of interventions (e.g., MDA, vector control) using transmission-dynamics, compartmental, stochastic, agent-based, or policy simulation models rather than predicting baseline disease risk.
- Focused exclusively on mathematical modelling of disease transmission without estimating individual-, community-, or spatial-level LF risk.
- Focused on animal or laboratory models.
- Were clinical studies, entomological surveys without a prediction modelling component, epidemiological descriptive studies, Knowledge, Attitudes and Practices (KAP) studies, case reports, commentaries, or methodological papers without original LF risk modelling.
- Did not relate specifically to LF or its causative parasites.

#### 2.3.3 Population and outcomes.

No restrictions were applied regarding study population or geographic location. Eligible studies included human populations from any LF-endemic setting. The primary predicted outcomes included LF prevalence, antigen positivity, microfilaria positivity, infection probability, environmental suitability, transmission risk, spatial risk, hotspot probability, or composite LF risk indices.

### 2.4 Data extraction

Data were extracted using a structured extraction form based on the CHARMS framework. Extracted information included study characteristics such as study design, data source, predictor domains, predictor selection strategies, modelling approaches, validation strategies, and outcome definitions. During data extraction, discrepancies that arose were resolved through discussion (S2 Material. Characteristics of included Lymphatic filariasis studies).

### 2.5 Risk of bias assessment

Three reviewers independently assessed the risk of bias of each included study using the PROBAST [10]. Risk of bias was assessed across the four PROBAST domains (participants, predictors, outcome, and analysis) using the corresponding signalling questions, and each domain was assigned a judgement of low, high, or unclear risk of bias. For the overall risk-of-bias judgement, a study was classified as having high risk when at least one PROBAST domain was judged as high or unclear risk. Any disagreements between reviewers were resolved through discussion until consensus was reached.

### 2.6 Data synthesis

Data were synthesised descriptively to summarise the characteristics of the included studies, including modelling approaches, predictor domains, outcome definitions, predictor selection methods, validation strategies, model performance measures, and risk-of-bias assessments. Where appropriate, methodological characteristics were also synthesised according to outcome type to examine differences in modelling approaches, predictor domains, validation strategies, and performance assessment across outcome categories. Quantitative summaries were used to describe the frequency and distribution of modelling characteristics across studies. Meta-analysis of predictive performance was not performed because of substantial methodological heterogeneity in study populations, outcome definitions, predictor sets, modelling approaches, validation methods, and performance measures. Consequently, publication bias and certainty of evidence were not formally assessed, as these methods are not appropriate in the absence of pooled quantitative estimates. Definitions of all methodological categories and coding rules are provided in S3 Material.

### 2.7 Ethical statement

This study is a systematic review of published literature and did not involve human participants, identifiable data, or primary data collection. Ethical approval was therefore not required.

## 3 Results

### 3.1 Study selection

The study selection process is presented in the PRISMA flow diagram (Fig 1). After removal of 2,412 duplicate records, 4,010 records underwent title screening, of which 1,393 records unrelated to LF were excluded. The remaining 2,617 records underwent abstract screening, resulting in the exclusion of 2,470 non-modelling studies. A total of 147 full-text articles were assessed for eligibility, of which 121 were excluded because they did not meet the predefined inclusion criteria. Ultimately, 26 studies met the eligibility criteria and were included in the qualitative and methodological synthesis. Key papers illustrating the range of modelling approaches and methodological characteristics identified in this review are highlighted in Box 2.

**Fig 1:**
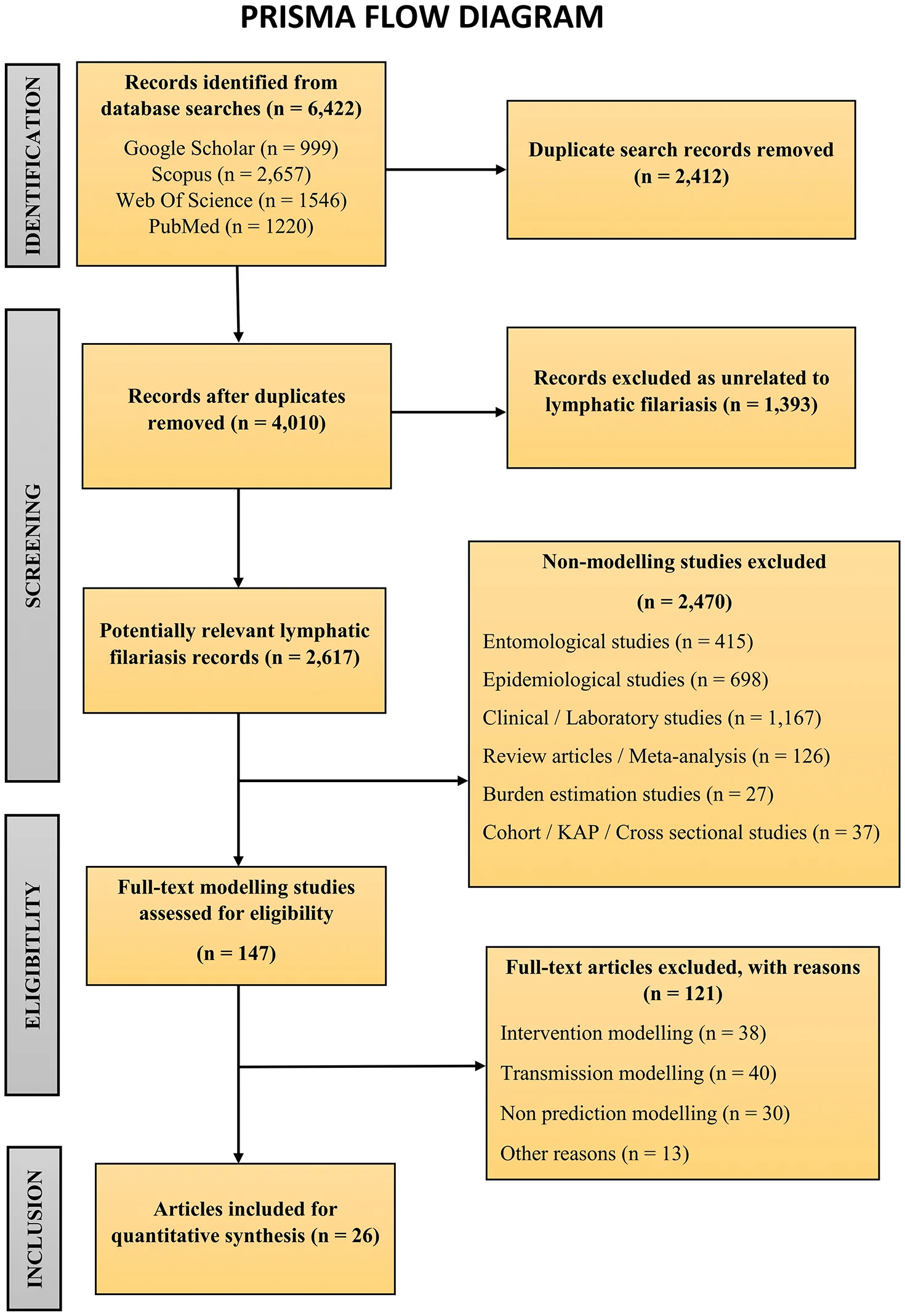
PRISMA flow diagram of the study selection process.

**Box 2 Key papers in the field**

**Table:** 

Study	Importance
Sabesan et al. 2013 [7]	LF transmission risk mapping (India)
Slater & Michael et al. 2013 [4]	Bayesian spatial modelling (Africa)
Mayfield et al. 2020 [8]	ML-based hotspot prediction (Samoa)
Eneanya et al. 2018 [14]	Environmental suitability modelling (Nigeria)
Stensgaard et al. 2011 [15]	Bayesian co-endemic modelling

### 3.2 Geographic distribution, study settings and data sources

The 26 included studies were published between 2000 and 2025 (Fig 2). National-level analyses were the most common geographic scale (11/26; 42.3%), followed by multi-country or regional (7/26; 26.9%) and sub-national studies (5/26; 19.2%) (Table 1).

**Table 1:** Sources of model data in LF risk prediction studies (n = 26).

Sources of model data	No. of Studies (%)*	Studies Reported
**LF epidemiological data source**		
Surveillance/notification data	9 (34.6%)	[14,16–23]
National disease registry/programme data	6 (23.1%)	[7,16,20,24–26]
Community surveys/field surveys	15 (57.7%)	[4,5,7,8,15,25,27–35]
**Environmental/climate data source**		
Government meteorological agencies	7 (26.9%)	[5,7,8,16,23,25,29]
Global climate datasets (e.g., WorldClim, CRU)	10 (38.5%)	[4,14,17–20,24,27,31,32]
Research institute–derived datasets	16 (61.5%)	[4,5,8,14–21,24,27,29,31,32]
**Vector data source**		
Entomological surveillance/monitoring	1 (3.8%)	[32]
**Population source**		
General population	22 (84.6%)	[4,5,7,8,14,16–19,21–24,26–32,34,35]
Specific sub-populations (communities/villages)	4 (15.4%)	[15,20,25,33]
**Geographic scale distribution**		
Multi-country/Regional	7 (26.9%)	[4,24,26,27,31,33,34]
National	11 (42.3%)	[7,8,14–21,30]
Sub-national	5 (19.2%)	[5,22,25,29,32]
District/Local Government Area	2 (7.7%)	[23,28]
Community/Village/Household	1 (3.8%)	[35]

Geographic scale distribution categories are mutually exclusive and based on the geographic extent of the study area (n = 26).

**Fig 2:**
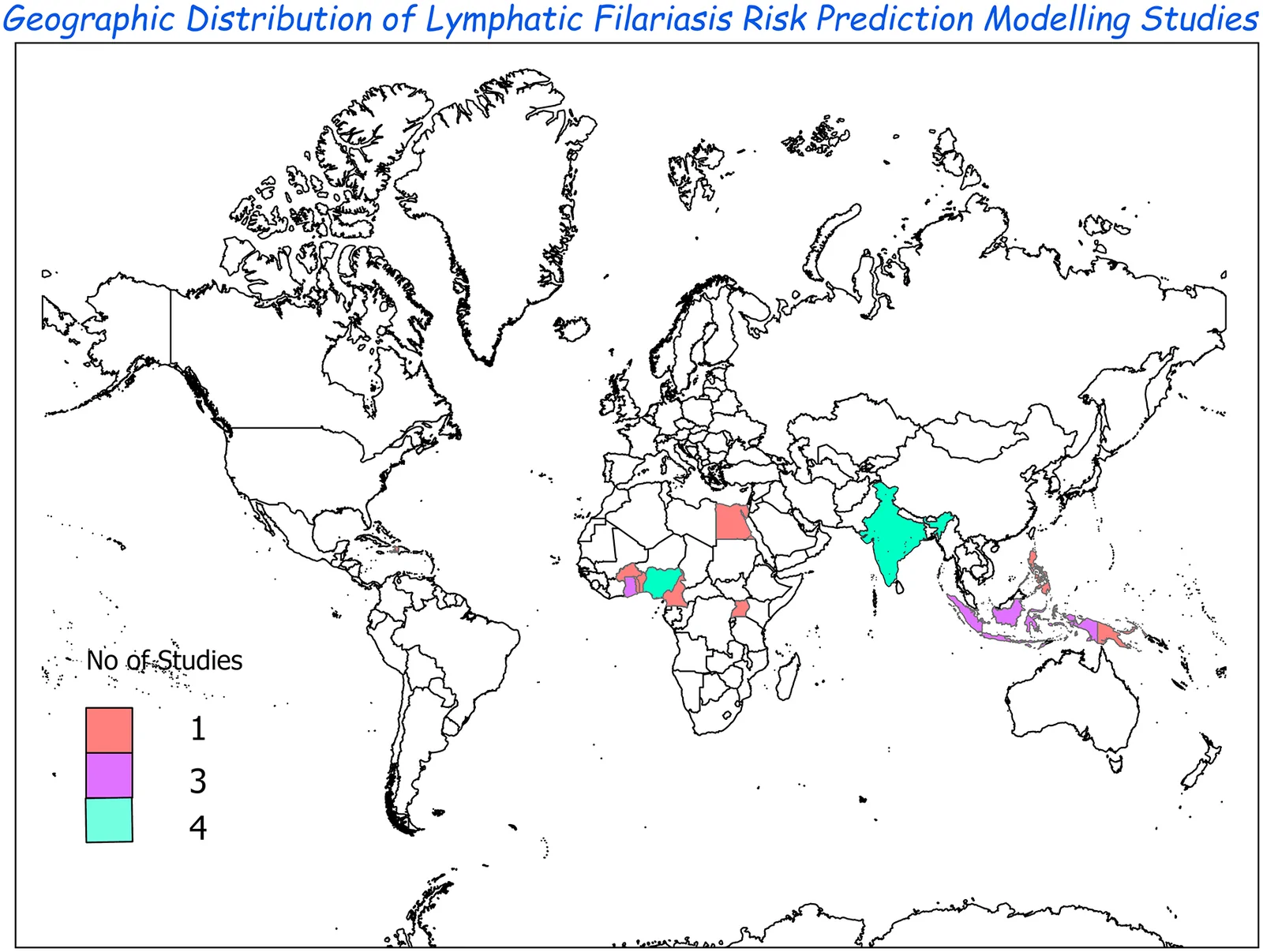
Geographic distribution of the included lymphatic filariasis risk prediction modelling studies. Base map derived from Natural Earth public domain vector data (https://www.naturalearthdata.com). The Indian international boundary follows the official representation of the Survey of India (S1 Fig).

Community or field surveys constituted the predominant source of LF epidemiological data (15/26; 57.7%). Environmental predictor data were most frequently obtained from research institute–derived datasets (16/26; 61.5%), followed by global climate datasets (10/26; 38.5%) and government meteorological agencies (7/26; 26.9%). Most studies were based on the general population (22/26; 84.6%), whereas entomological surveillance data were incorporated in only one study (1/26; 3.8%) (Table 1).

### 3.3. Predictor domains and selection strategies

Predictors were drawn from multiple domains (Tables 2 and S2). Environmental predictors were the most frequently incorporated domain (20/26; 76.9%), followed by climatic predictors (19/26; 73.1%) and socio-demographic predictors (15/26; 57.7%). Programmatic or MDA history variables and entomological predictors were incorporated in only two studies each (2/26; 7.7%). Detailed distributions of individual predictor variables across studies are presented in S2 Table.

**Table 2:** Modelling strategies and validation practices in LF risk prediction studies (n = 26).

Domain	Category	No. of Studies (%)*	Studies Reported
**Predictor domains**	Climatic factors	19 (73.1%)	[4,5,7,8,14–20,23–25,27,29,31,32,34]
	Environmental factors	20 (76.9%)	[4,5,7,8,14–21,23–27,29,31,32]
	Socio-demographic factors	15 (57.7%)	[4,5,15–20,22–24,27–29,35]
	Entomological (vector) factors	2 (7.7%)	[21,24]
	Programmatic/MDA history	2 (7.7%)	[30,35]
**Predictor selection strategies**	Expert-driven inclusion	11 (42.3%)	[7,16,20–23,25,26,30,31,34]
	Univariate screening	2 (7.7%)	[5,35]
	Algorithm-based selection	12 (46.2%)	[4,8,14,15,17–19,24,27–29,32]
**Modelling approaches**	Bayesian geo-statistical/spatial	10 (38.5%)	[4,15,16,20–22,24,29,33,35]
	Conventional statistical models	6 (23.1%)	[5,7,25,26,30,34]
	Machine learning models	4 (15.4%)	[14,18,19,28]
	Ecological niche modelling (ENM)	2 (7.7%)	[14,27]
	Hybrid/ensemble approaches	3 (11.5%)	[8,20,31]
	Alternative modelling approaches	3 (11.5%)	[23,32,35]
**Validation strategies**	Internal validation	13 (50.0%)	[4,8,14,15,17–19,24,27–29,31,34]
	External validation	4 (15.4%)	[7,25,26,33]
	Partial/limited validation	3 (11.5%)	[4,26,32]
	No validation reported	8 (30.8%)	[5,16,20–23,30,35]
**Validation techniques reported**	Cross-validation	6 (23.1%)	[17–19,24,28,31]
	Split-sample validation	5 (19.2%)	[14,15,27,29,34]
	Retrospective validation	1 (3.8%)	[8]
	Performance metrics only	14 (53.8%)	[4,5,7,16,20–23,25,26,30,32,33,35]
**Performance metrics reported**	Discrimination (AUC/ROC)	5 (19.2%)	[7,14,18,27,29]
	Accuracy/error metrics (RMSE, MAE, MAPE)	19 (73.1%)	[4,5,8,14,15,17–22,24–26,28,30–32,34]
	Qualitative/visual assessment only	6 (23.1%)	[8,16,26,32,33,35]
	Calibration metrics	2 (7.7%)	[7,25]

* Percentages may not sum to 100 as studies used multiple methods and Validation strategy categories are not mutually exclusive; a study may report both internal and partial/limited validation simultaneously.

Among climatic predictors, temperature and rainfall were the most frequently used variables (18/26; 69.2% each), whereas altitude or topography (17/26; 65.4%) and vegetation indices (NDVI/EVI) (9/26; 34.6%) were the most common environmental predictors. Population density was the most frequently reported socio-demographic predictor (10/26; 38.5%), while socioeconomic proxies, including housing, sanitation, education, and MDA participation, were reported in 9/26 studies (34.6%) (S2 Table).

Predictor selection strategies were reported in 24 studies. Algorithm-based selection methods were the most common (12/26; 46.2%), followed by expert-driven inclusion (11/26; 42.3%), whereas univariate screening was reported in only two studies (7.7%) (Table 2).

### 3.4. Modelling approaches

Bayesian geo-statistical and spatial models were the most frequently used modelling approaches, reported in 10/26 studies (38.5%). Conventional statistical models, including logistic and other regression-based approaches, were used in 6/26 studies (23.1%). ML methods were applied in 4/26 studies (15.4%), whereas ENM approaches were reported in 2/26 studies (7.7%). Hybrid or ensemble approaches were identified in 3/26 studies (11.5%), and three additional studies employed alternative modelling approaches, including GIS-based weighted overlay scoring, descriptive spatial interpolation, and hotspot analysis (Table 2).

### 3.5. Outcome definitions, validation strategies and model performance

Outcome definitions varied across the included studies (Table 3). Continuous infection prevalence was the most frequently modelled outcome (11/26; 42.3%), followed by binary infection or endemicity status (6/26; 23.1%), composite risk or vulnerability classification (5/26; 19.2%), case burden or counts (2/26; 7.7%), and environmental or vector suitability (2/26; 7.7%) (Table 3). These outcome categories represent distinct modelling objectives and were therefore interpreted separately.

**Table 3:** Outcome-stratified methodological characteristics of lymphatic filariasis risk prediction studies.

Outcome category	No. of studies (%)	Studies reported	Predictor domains	Modelling approaches	Validation strategy	Validation technique	Performance assessment
Binary Infection/Endemicity Status	6 (23.1%)	[7,18,25,28,29,34]	Climatic (5); Environmental (4); Socio-demographic (3)	Machine learning (2); Conventional statistical (3); Bayesian/spatial (1)	Internal (4); External (2)	Cross-validation (2); Performance metrics only (2); Split-sample (2)	Discrimination/AUC (3); Accuracy/error (4); Calibration (2)
Case Burden/Counts	2 (7.7%)	[20,22]	Climatic (1); Environmental (1); Socio-demographic (2)	Bayesian/spatial (2); Hybrid/ensemble (1)	None reported	Performance metrics only (2)	Accuracy/error (2)
Composite Risk/Vulnerability Classification	5 (19.2%)	[16,23,31,32,35]	Climatic (4); Environmental (4); Socio-demographic (3); MDA/programmatic (1)	Bayesian/spatial (2); Hybrid/ensemble (1); Other models (3)	Internal (1); Partial/limited (1)	Cross-validation (1); Performance metrics only (4)	Accuracy/error (2); Qualitative/visual (3)
Environmental/Vector Suitability	2 (7.7%)	[14,27]	Climatic (2); Environmental (2); Socio-demographic (1)	ENM (2)	Internal (2)	Split-sample (2)	Discrimination/AUC (2); Accuracy/error (1)
Infection Prevalence (continuous)	11 (42.3%)	[4,5,8,15,17,19,21,24,26,30,33]	Climatic (7); Environmental (9); Socio-demographic (6); MDA/programmatic (1); Vector-related (2)	Bayesian/spatial (5); Conventional statistical (3); Machine learning (2); Hybrid/ensemble (1)	Internal (6); External (2); Partial/limited (2)	Cross-validation (3); Performance metrics only (6); Split-sample (1); Retrospective (1)	Accuracy/error (10); Qualitative/visual (3);

Internal validation was reported in 13/26 studies (50.0%), whereas external validation was reported in 4/26 studies (15.4%). All four externally validated models used geographically independent validation data, including newly surveyed sites, previously unsurveyed districts or communities, or new villages in different geographic settings, rather than temporally independent data; none reported temporal external validation across distinct epidemiological or programme periods (S4 Table). Partial or limited validation was identified in 3/26 studies (11.5%). Cross-validation was the most frequently reported validation technique (6/26; 23.1%), followed by split-sample validation (5/26; 19.2%) and retrospective validation (1/26; 3.8%). Fourteen studies (53.8%) reported performance metrics without specifying a distinct validation technique (Table 2).

Model performance reporting was heterogeneous. Accuracy or error-based metrics (e.g., RMSE, MAE, and MAPE) were the most frequently reported measures (19/26; 73.1%), followed by qualitative or visual assessment (6/26; 23.1%) and discrimination metrics such as the area under the receiver operating characteristic curve (AUC/ROC) (5/26; 19.2%). Formal calibration assessment was reported in only two studies (2/26; 7.7%) (Table 2).

Outcome-stratified methodological characteristics are presented in Table 3. Continuous infection prevalence models showed the greatest diversity of modelling approaches, including Bayesian/spatial (5/26), conventional statistical (3/26), machine learning (2/26), and hybrid/ensemble (1/26) approaches, with environmental predictors reported most frequently (9/26). Binary infection/endemicity models included conventional statistical (3/26), machine-learning (2/26), and Bayesian/spatial (1/26) approaches, with both internal and external validation represented. Environmental/vector suitability models exclusively used ecological niche modelling (2/26) with split-sample validation (2/26). Composite risk/vulnerability models showed heterogeneous modelling approaches, while both case burden/count models used Bayesian/spatial approaches and reported no formal validation. Formal calibration assessment was restricted to binary infection/endemicity models, while qualitative/visual assessment was most prominent among composite risk/vulnerability models. Across outcome categories, climatic and environmental predictors were the most consistently represented domains, whereas vector-related and MDA/programmatic predictors were less frequently incorporated (Table 3). Detailed study-level methodological characteristics are provided in S4 Table.

### 3.6. Methodological transparency and translational characteristics

Methodological transparency and translational characteristics of the included studies are summarised in Table 4. Uncertainty quantification and measures to minimise overfitting were reported in 15/26 (57.7%) studies each. Explicit assessment or management of predictor collinearity was reported in 4/26 (15.4%) studies. Missing-data handling was described in 7/26 (26.9%) studies, whereas formal calibration assessment was reported in only 2/26 (7.7%) studies. Temporal alignment between predictor and outcome data was variably reported across studies. Twelve studies (46.2%) explicitly described contemporaneous or temporally matched predictor and outcome data, whereas seven (26.9%) relied predominantly on long-term or historical predictor averages. Two studies (7.7%) described partial or attempted temporal matching, one study (3.8%) explicitly acknowledged temporal mismatch, and four studies (15.4%) did not clearly report predictor timing. Study-level classifications and supporting details are provided in S5 Table. Evidence of implementation within routine public health or national LF programmes was identified in 1/26 (3.8%) study. Overall, reporting of methodological and implementation-related characteristics was inconsistent across the included studies (Table 4).

**Table 4:** Methodological Robustness, Transparency, and Translational Characteristics of Included lymphatic filariasis Risk Prediction Models (n = 26).

Characteristics	No. of Studies (%)	Studies Reported
**Methodological Transparency & Rigor**		
Uncertainty quantification	15 (57.7%)	[4,5,7,8,14–17,19–21,24,26,27,29]
Formal Calibration Plot Assessment	2 (7.7%)	[7,25]
Code/Script Availability	0 (0.0%)	
Missing data handling reported	7 (26.9%)	[4,14,17,19–21,27]
Overfitting control reported	15 (57.7%)	[7,8,14,15,17–19,24–29,31,34]
collinearity assessment/management	4 (15.4%)	[4,14,18,27]
**Translational Readiness**		
Government/Policy Integration	1 (3.8%)	[20]
Model Operationalisation	0 (0.0%)	
Early Warning/Surveillance Integration	0 (0.0%)	

### 3.7. Cross-study methodological patterns

Joint examination of predictor domains, modelling approaches, validation strategies, and validation techniques demonstrated recurring methodological co-occurrence patterns across the included studies (Fig 3).

**Fig 3:**
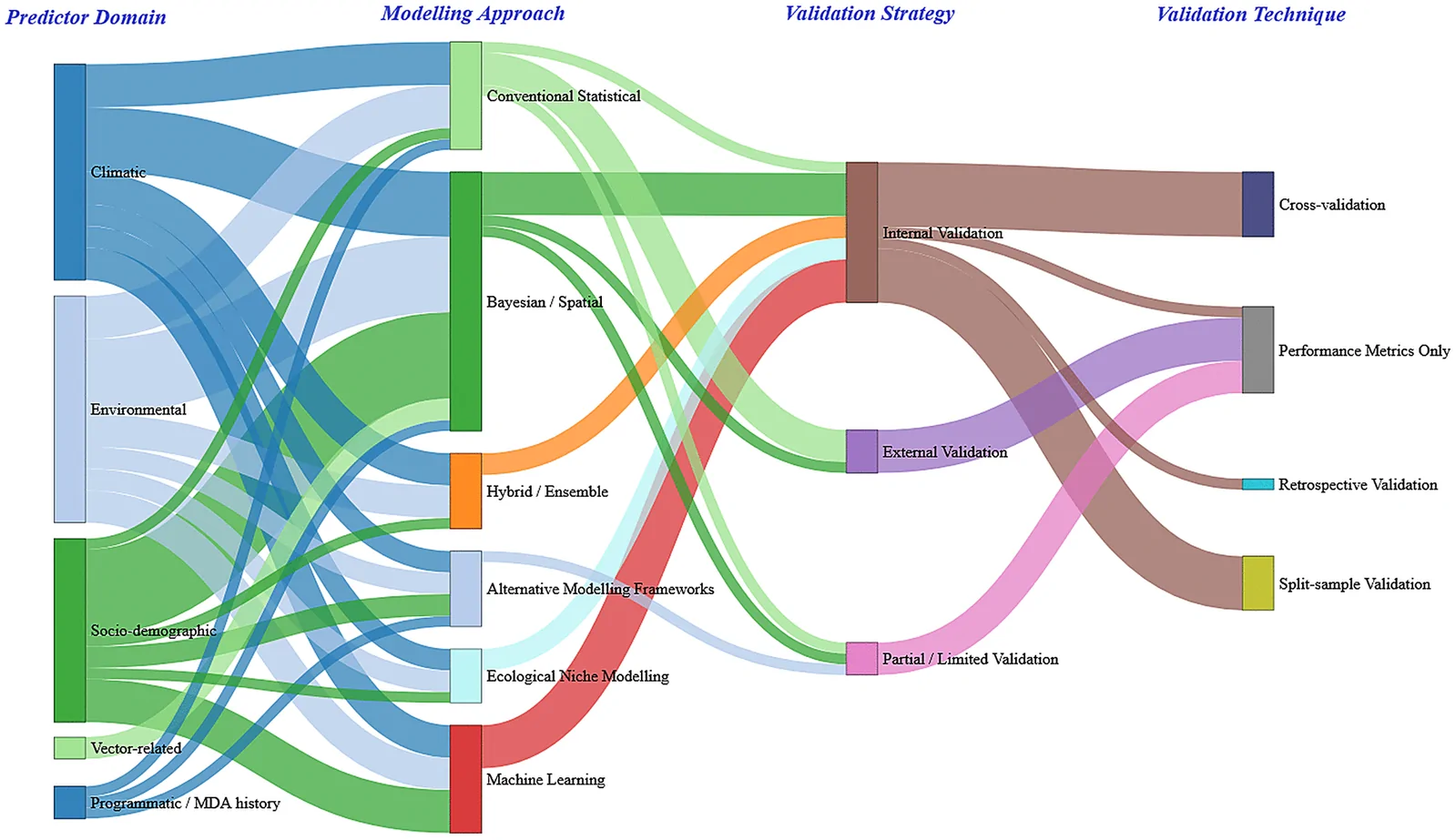
Structural alignment of predictor domains, modelling approaches, validation strategies, and validation techniques across lymphatic filariasis risk prediction studies (S1 Table and S2 Fig).

Climatic and environmental predictor domains showed substantial overlap across modelling approaches, with Bayesian/spatial and conventional statistical models receiving the largest number of connections from these predictor domains. Socio-demographic predictors were incorporated across multiple modelling approaches, whereas vector-related and programmatic/MDA predictors were represented in comparatively fewer studies (Fig 3).

Distinct co-occurrence patterns were also observed between modelling approaches and validation strategies. Bayesian/spatial, conventional statistical, and ML models were predominantly linked to internal validation, whereas external and partial validation were reported for a smaller number of modelling approaches. Hybrid/ensemble, ecological niche, and alternative modelling frameworks were represented less frequently across validation strategies (Fig 3).

Internal validation was most commonly associated with cross-validation and split-sample validation techniques, while performance metrics without explicit data splitting were reported across internal, external, and partial validation strategies. Retrospective validation was reported infrequently (Fig 3).

Pairwise associations between methodological characteristics are summarised in S2 Fig. Positive associations were observed between several methodological characteristics, including climatic and environmental predictor domains, ML models and cross-validation, and algorithm-based predictor selection with internal validation. Negative associations were identified between several mutually exclusive methodological characteristics, including expert-driven versus algorithm-based predictor selection and internal validation versus performance metrics without explicit validation (S2 Fig).

### 3.8. Risk of bias assessment

Risk of bias was assessed using the PROBAST tool. Domain-level judgements are summarised in Table 5, study-level assessments are presented in S3 Table, and question-level responses to all PROBAST signalling questions are provided in S4 Material.

**Table 5:** Risk of bias assessment of included LF risk prediction studies using PROBAST (n = 26).

PROBAST domain	Low risk n (%)	High/Unclear risk n (%)*
Participants	24 (92.3)	2 (7.7)
Predictors	21 (80.8)	5 (19.2)
Outcome	26 (100.0)	0 (0.0)
Analysis	0 (0.0)	26 (100.0)**
Overall risk of bias	0 (0.0)	26 (100.0)***

*High/Unclear risk combines studies judged as having high or unclear risk of bias.

**In the analysis domain, 11 studies (42.3%) were judged as high risk and 15 (57.7%) as unclear risk.

***Overall risk of bias was classified as high when at least one PROBAST domain was judged as high or unclear risk.

Most studies were judged to have a low risk of bias in the participants domain (24/26; 92.3%), whereas two studies (7.7%) were classified as having high or unclear risk. In the predictors domain, 21 studies (80.8%) were judged to have a low risk of bias, while five studies (19.2%) were classified as having high or unclear risk. All included studies were judged to have a low risk of bias in the outcome domain (26/26; 100.0%).

In contrast, all studies were judged to have high or unclear risk of bias in the analysis domain (26/26; 100.0%), comprising 11 studies at high risk and 15 at unclear risk. As a result, all included studies were classified as having high overall risk of bias (26/26; 100.0%) (Table 5). Question-level PROBAST assessments indicated that concerns in the analysis domain were particularly related to inadequate or insufficiently reported sample size/events-per-variable information, with 23/26 studies (88.5%) rated unclear for this signalling question. Concerns regarding overfitting and validation procedures were also identified, with 8/26 studies (30.8%) rated no for this signalling question (S4 Material).

## 4. Discussion

### 4.1. Importance of LF risk prediction modelling in the elimination and post-elimination era

This review provides the first methodological synthesis of LF risk prediction models using established prediction-model appraisal frameworks. Prediction modelling for LF presents unique challenges compared with many acute infectious diseases because infection reflects cumulative exposure over prolonged periods and is strongly influenced by historical intervention activities, particularly repeated rounds of MDA. Consequently, climatic and environmental variables alone are unlikely to adequately characterise contemporary transmission risk, especially in elimination settings where observed infection patterns increasingly reflect the combined effects of past transmission, programme implementation, and long-term ecological conditions. Robust LF risk prediction therefore requires integration of epidemiological, environmental, socio-demographic, programmatic, and, where available, entomological data, together with modelling approaches capable of accounting for complex spatial heterogeneity.

These characteristics have important implications for the use of prediction models in LF elimination programmes. As countries progress towards elimination verification and post-elimination surveillance, prediction models are increasingly expected to support surveillance prioritisation and identification of residual transmission foci. However, the predominance of internally validated models observed in this review suggests that the generalisability of many published models beyond their development settings remains uncertain. Prediction models intended to inform surveillance and resource allocation should therefore demonstrate not only good predictive performance but also adequate transportability across different epidemiological, environmental, and programmatic settings [10,13].

Unlike previous LF modelling literature, which has largely focused on transmission dynamics or evaluation of intervention strategies [6,36], this review specifically evaluated the methodological characteristics of LF risk prediction models, including predictor selection, model development, validation strategies, performance assessment, and risk of bias. The findings highlight the need for more rigorous external validation, comprehensive performance evaluation, and transparent reporting to improve confidence in the application of prediction models for surveillance and evidence-based decision-making.

### 4.2. Geographic distribution, study settings, and data availability

The predominance of studies conducted in Africa likely reflects the continued public health importance of LF in the region and the availability of surveillance data to support predictive modelling [2,3]. Nevertheless, the geographic distribution of studies remained uneven, with comparatively limited evidence from endemic regions in the Americas and the Western Pacific [37]. Broader geographic representation will be important for improving the generalisability and transferability of future LF risk prediction models.

The predominance of national-level analyses observed in this review suggests that model development has largely been driven by the availability of routinely collected surveillance data and spatially continuous environmental datasets. Freely available geospatial products, including WorldClim and Climatic Research Unit (CRU) datasets, have facilitated large-scale spatial prediction by providing consistent environmental information across broad geographic regions [38,39]. In contrast, comparatively few studies were conducted at community or village level, indicating that limited availability of high-resolution epidemiological data continues to constrain fine-scale risk prediction. The predominance of spatially aggregated datasets also raises the possibility of ecological bias, whereby associations observed using area-level predictors may not accurately reflect relationships at the individual level [40].

The widespread use of research institute-derived environmental datasets further highlights the growing importance of remotely sensed and geospatial data in LF risk prediction. However, only one study has incorporated entomological surveillance data despite the recognised contribution of vector ecology to LF transmission. Greater integration of epidemiological, environmental, entomological, demographic, and programmatic datasets may improve the ecological relevance and spatial precision of future prediction models, particularly for identifying residual transmission foci during elimination and post-elimination surveillance.

Overall, these findings indicate that current LF risk prediction models remain strongly influenced by the availability and spatial resolution of underlying datasets. Improving access to routinely collected high-resolution surveillance, entomological, and programme data will be essential for developing more robust and operationally relevant prediction models capable of supporting targeted surveillance and evidence-based decision-making.

### 4.3. Interpretation of predictor domains and selection strategies

The predominance of environmental and climatic predictors observed in this review reflects both their established role in shaping LF transmission and the widespread availability of spatially continuous environmental datasets. Variables such as temperature, rainfall, altitude, and vegetation indices are routinely available through remote-sensing platforms and global geospatial databases, facilitating their integration into spatial prediction models [5,38,39]. Their extensive use across modelling approaches suggests that these predictors currently form the methodological foundation of LF risk prediction.

In contrast, socio-demographic, programmatic, and entomological predictors were incorporated less consistently despite their recognised contribution to LF transmission. The limited inclusion of programmatic variables, including MDA history, and the infrequent use of entomological data likely reflect challenges in obtaining standardised, spatially resolved datasets rather than limited epidemiological importance [4,6]. Greater integration of epidemiological, environmental, entomological, and programme data may improve the ability of future models to capture the multifactorial nature of LF transmission, particularly in low-transmission and post-elimination settings.

The variation in predictor selection strategies across modelling frameworks reflects differences in modelling philosophy rather than a standardised approach to variable selection. Biologically informed approaches were more common in Bayesian and spatial models, whereas algorithm-based selection predominated in ML models. Regardless of the approach used, transparent reporting of predictor selection procedures is essential because variable selection methods can influence model reproducibility, comparability, and transportability across different epidemiological settings [9,41].

### 4.4. Evolution of LF risk prediction models

The temporal distribution of modelling approaches indicates a gradual diversification of analytical methods rather than a replacement of established modelling frameworks (Fig 4). Earlier studies predominantly employed conventional statistical models and Bayesian geo-statistical approaches, reflecting the availability of surveillance data and advances in spatial epidemiological modelling [24,42]. More recent studies have incorporated ML, ENM, hybrid, and other alternative modelling approaches, expanding the range of analytical tools available for LF risk prediction.

**Fig 4:**
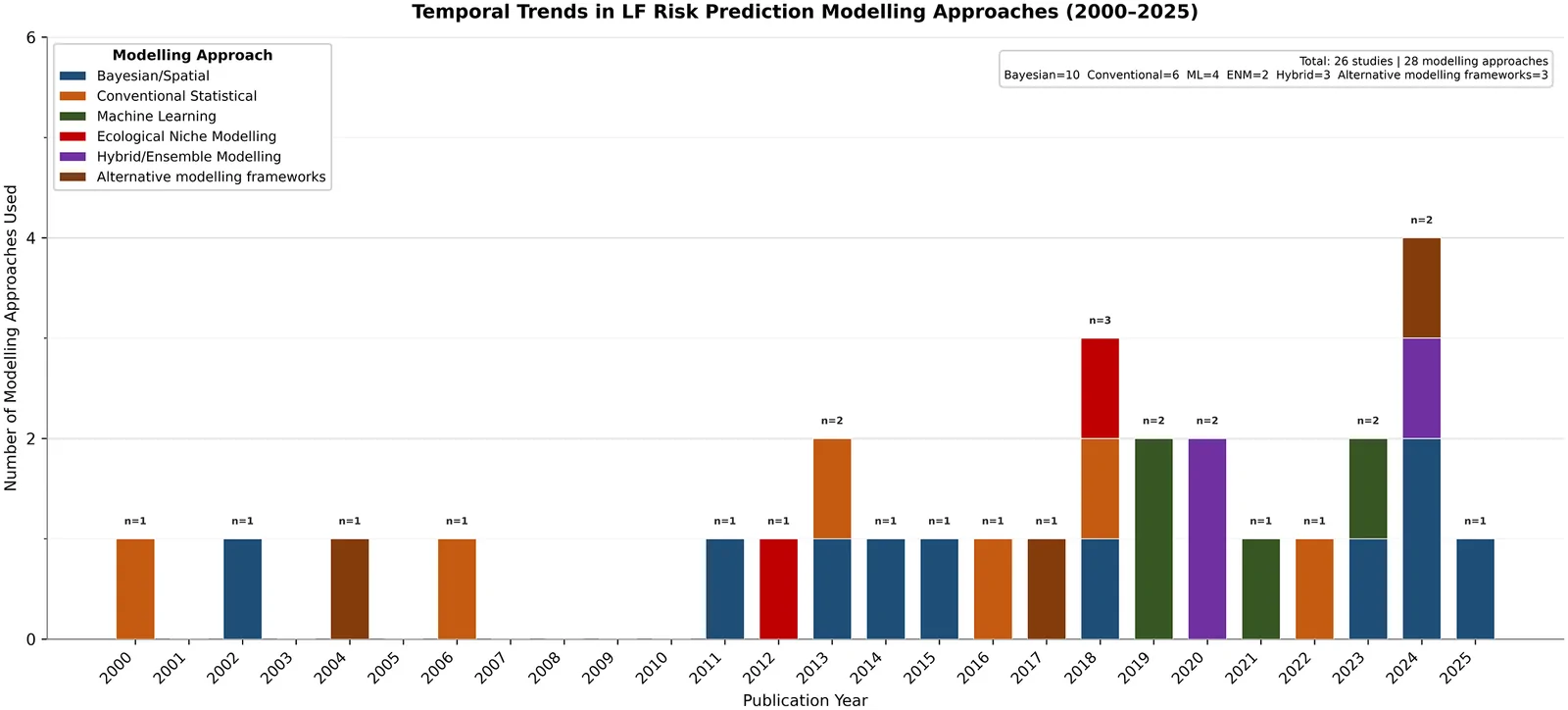
Temporal trends LF risk prediction modelling approaches.

Despite this diversification, the continued prominence of Bayesian and spatial modelling approaches reflects their suitability for modelling the heterogeneous spatial distribution of LF. By explicitly accounting for spatial dependence and uncertainty, these models generate interpretable risk estimates that are well suited to disease mapping and surveillance applications [24,42]. In contrast, ML and hybrid approaches offer greater flexibility for modelling complex, nonlinear relationships among environmental and demographic predictors, particularly where high-dimensional datasets are available [41]. Rather than replacing established spatial modelling frameworks, these approaches appear to complement existing analytical methods by expanding the range of modelling tools available for LF risk prediction.

### 4.5. Validation practices and performance reporting

Internal validation was the predominant approach across studies, most frequently implemented through cross-validation or split-sample validation techniques. Although these methods provide an assessment of model stability within the development dataset, they do not establish whether a model will maintain its performance when applied to different populations, geographical settings, or stages of LF elimination. The limited use of external validation observed in this review is consistent with findings from broader prediction modelling literature, where independent evaluation of model transportability remains uncommon [9,43].

Among the four studies that reported external validation, validation was consistently performed using geographically independent populations or survey locations rather than temporally independent datasets. Geographic validation provides stronger evidence of model transportability than internal validation alone because model performance is evaluated in populations that were not used during model development. However, none of the included studies assessed temporal external validation across different transmission periods or programme phases. Consequently, evidence regarding the temporal stability of published LF risk prediction models remains limited, particularly in the context of changing epidemiological conditions during elimination and post-elimination surveillance.

Model calibration was infrequently assessed despite being a fundamental component of prediction model evaluation. Calibration reflects the agreement between predicted and observed outcomes and determines whether predicted risks are clinically and programmatically meaningful [44]. The limited reporting of calibration observed in this review suggests that the reliability of absolute risk estimates remains uncertain for many published LF prediction models. In elimination settings, inaccurate risk estimates may lead to over- or under-prioritisation of surveillance activities and resource allocation. For example, a poorly calibrated model predicting 30% antigen prevalence in a district with true prevalence of 10% could lead programme managers to unnecessarily implement additional MDA rounds or delay elimination verification.

Performance evaluation also varied according to the modelling objective and outcome definition. Continuous outcomes were generally assessed using error-based measures such as RMSE, MAE, or MAPE, whereas binary outcomes more frequently employed discrimination measures such as the area under the receiver operating characteristic curve (AUC/ROC). Studies generating spatial risk surfaces often relied on qualitative or visual assessment. Although these evaluation approaches are appropriate for different prediction objectives, the lack of greater consistency in performance reporting limits direct comparison across studies and makes objective evaluation of relative model performance more difficult.

### 4.6. Methodological transparency and translation into practice

Methodological transparency remains an important challenge in LF risk prediction research. Explicit assessment or management of predictor collinearity was uncommon, despite the potential for correlation among climatic, environmental, and socio-demographic predictors. Limited reporting of missing-data handling and formal calibration assessment, together with inconsistent documentation of model development procedures and the complete absence of publicly available analytical code or model scripts, reduces the reproducibility and interpretability of published prediction models [43,45]. Among the studies reporting missing-data handling, approaches included complete-case analysis, exclusion of incomplete observations, and model-specific handling procedures, their reporting detail was generally insufficient to evaluate their impact. Temporal alignment between predictor and outcome data was also inconsistently reported, with some studies using contemporaneous or matched predictor data and others relying on long-term climatological averages or predictor datasets from different periods. Although uncertainty quantification and measures to minimise overfitting were reported in more than half of the included studies, greater adherence to established methodological guidance would improve the transparency and reliability of future prediction models [9,10].

Despite the increasing application of LF risk prediction models, evidence of their implementation within routine public health programmes was limited. While many studies generated spatial risk maps or predictive risk estimates, few described integration into national LF surveillance systems or programme decision-making. As countries progress towards elimination and post-elimination surveillance, greater emphasis on external validation, transparent reporting, and evaluation under operational conditions will be important to facilitate the translation of prediction models into routine public health practice.

Successful implementation also depends on practical considerations beyond methodological quality. Bayesian geo-statistical models often require specialised software and substantial computational resources [46], while routine surveillance systems in many endemic settings lack automated workflows that integrate epidemiological, environmental, and programme data for prediction modelling. Strengthening data infrastructure, computational capacity, and integration of surveillance systems may therefore be as important as methodological improvements for supporting the routine use of LF risk prediction models.

### 4.7. Cross-methodological structural patterns

The methodological co-occurrence patterns observed in this review suggest that prediction model development in LF is characterised by established analytical workflows rather than independent methodological choices. Predictor selection, modelling framework, validation strategy, and performance evaluation appeared to be closely interconnected, indicating that these components are often selected as part of a broader modelling paradigm rather than optimised individually. Such clustering likely reflects both the characteristics of available datasets and the methodological conventions associated with different analytical approaches.

From a methodological perspective, these structured relationships highlight the importance of considering prediction model development as an integrated process. The reliability of a prediction model depends not only on the choice of modelling algorithm but also on the coherence between predictor selection, outcome definition, validation strategy, and performance assessment. Consequently, future LF risk prediction studies should evaluate these methodological components collectively rather than in isolation and report them transparently in accordance with established prediction modelling guidance [9,41].

### 4.8. Outcome heterogeneity and comparability

This review identified substantial heterogeneity in outcome definitions across LF risk prediction studies, with models predicting continuous infection prevalence, binary infection or endemicity status, case burden, environmental suitability, and composite risk classifications. Differences in diagnostic approaches, including microfilaraemia microscopy and circulating filarial antigen detection, further contributed to variation in the predicted outcomes.

The methodological characteristics of the models also varied according to outcome type. Models predicting continuous infection prevalence encompassed a broad range of modelling approaches, whereas environmental or vector suitability models consistently used ecological niche modelling. Binary infection or endemicity models incorporated both statistical and machine-learning approaches, while composite risk or vulnerability models showed greater methodological heterogeneity. These differences indicate that the choice of modelling framework was closely linked to the nature and intended use of the predicted outcome rather than representing a single standardised approach to LF risk prediction.

Such heterogeneity limits direct comparison of model performance across studies. Different outcome definitions, measurement procedures, and modelling objectives require different modelling frameworks and evaluation metrics, making a common interpretation of measures such as accuracy, error, discrimination, or calibration difficult. This heterogeneity, together with variation in validation practices, was therefore an important reason why quantitative synthesis of model performance was not considered appropriate in the present review [13,41].

From a programme perspective, the distinction between outcome types is particularly relevant because model outputs may serve different purposes, including surveillance, identification of residual transmission foci, estimation of infection burden, assessment of environmental suitability, or prioritisation of interventions. Greater consistency in defining and reporting prediction outcomes, together with transparent description of diagnostic ascertainment and validation procedures, would improve comparability across studies and facilitate external validation and implementation in different epidemiological settings.

### 4.9. Risk of bias

Application of the PROBAST framework demonstrated that all included studies were judged to have a high overall risk of bias, primarily because of limitations within the analysis domain. In contrast, most studies were assessed as having a low risk of bias in the participants, predictors, and outcome domains, indicating that the principal methodological weaknesses were related to model development and evaluation rather than study populations or outcome measurement [10].

The consistently high risk of bias in the analysis domain reflected recurrent methodological limitations identified across the included studies, including limited external validation, infrequent assessment of calibration, incomplete reporting of model development procedures, insufficient evaluation of overfitting, and inconsistent reporting of missing-data handling. These shortcomings reduce confidence in the reproducibility and transportability of published prediction models, particularly when applied beyond the populations or settings in which they were originally developed [9,10].

From a public health perspective, these methodological limitations are particularly relevant in elimination and post-elimination settings, where prediction models may be used to support surveillance prioritisation and resource allocation. Improving adherence to established guidance for prediction model development, validation, and reporting will therefore be essential for increasing confidence in future LF risk prediction models and facilitating their translation into routine programme decision-making [9,41].

### 4.10. Implications for future LF risk prediction research

This review identifies several priorities for strengthening future LF risk prediction research. Greater consistency in outcome definitions would improve comparability across studies and facilitate cumulative methodological development. Increased transparency in predictor selection, model development procedures, and reporting would enhance the reproducibility of published models. More comprehensive performance evaluation, including assessment of calibration in addition to discrimination and error-based metrics, would improve the interpretability and practical applicability of model outputs.

Future studies should place greater emphasis on external validation using geographically and temporally independent populations to improve model generalisability. As LF programmes transition towards elimination and post-elimination surveillance, prediction models are likely to play an increasingly important role in supporting targeted surveillance and resource allocation. Adherence to established methodological and reporting guidance, including TRIPOD and PROBAST, will strengthen the transparency, credibility, and policy relevance of future LF risk prediction models [9,10,45].

Several limitations of this review should be acknowledged. First, the review was not prospectively registered in PROSPERO, which may have reduced transparency regarding protocol amendments during the review process. Second, although study selection and data extraction were performed independently by three reviewers with disagreements resolved through consensus, inter-reviewer agreement statistics (e.g., Cohen’s κ) were not calculated and therefore reviewer agreement could not be quantitatively assessed. Third, only peer-reviewed studies published in the English language were included, potentially excluding relevant evidence reported in other languages, grey literature, and operational programme reports. Although multiple bibliographic databases were searched using comprehensive search strategies, indexing bias cannot be excluded. Finally, publication bias was not formally assessed because the substantial methodological heterogeneity of the included studies and the absence of pooled quantitative effect estimates precluded meaningful evaluation.

## 5. Conclusion

This systematic review provides the first comprehensive methodological synthesis of LF risk prediction models. Although modelling approaches have diversified considerably over the past two decades, important methodological limitations remain. Environmental and climatic predictors continue to dominate model development, whereas socio-demographic, entomological, and programmatic factors are incorporated less consistently. Furthermore, limited external validation, infrequent calibration assessment, and high or unclear risk of bias in the analysis domain reduce confidence in the generalisability and operational applicability of many published models.

As countries progress towards elimination and post-elimination surveillance, robust risk prediction models have the potential to support targeted surveillance, prioritisation of residual transmission foci, and more efficient allocation of programme resources. Achieving this potential will require greater methodological transparency, improved external validation across diverse epidemiological settings, standardised outcome definitions, and adherence to established prediction modelling and reporting guidance. Strengthening these methodological foundations will enhance the reliability, reproducibility, and policy relevance of future LF risk prediction models and support evidence-based decision-making for the long-term elimination of LF.

### Patient public involvement

No patients or public were involved in this study.

## Supporting information

S1 Table Study-level matrix of predictor domains, modelling approaches, and validation practices in lymphatic filariasis risk prediction studies. (DOCX)

S2 Table Predictor domains used in lymphatic filariasis risk prediction models. (DOCX)

S3 Table Study-level PROBAST risk of bias assessment for included lymphatic filariasis risk prediction studies. (DOCX)

S4 Table Study-level outcome and methodological characteristics. (DOCX)

S5 Table Study-level assessment of temporal alignment between predictor and outcome data. (DOCX)

S1 Fig Year-wise Distribution of LF risk prediction modelling studies. (TIF)

S2 Fig Pairwise co-occurrence of methodological characteristics across lymphatic filariasis risk prediction studies. (TIF)

S1 Material Detailed search strategies used for identification of lymphatic filariasis risk prediction modelling studies. (DOCX)

S2 Material Characteristics of included lymphatic filariasis risk prediction modelling studies. (XLSX)

S3 Material Classification framework used for methodological data extraction. (DOCX)

S4 Material Question-level responses to the PROBAST signalling questions for all domains across the included lymphatic filariasis risk prediction modelling studies. (XLSX)

S5 Material PRISMA 2020 checklist for the systematic review of lymphatic filariasis risk prediction modelling studies. (DOCX)

S6 Material Complete list of the 6,422 records identified through the literature search for the systematic review of lymphatic filariasis risk prediction modelling studies. (XLSX)
