# Supplementary material for: Lymphatic filariasis risk prediction models: A systematic review of modelling approaches, predictors, validation practices, and methodological quality
Source: PLoS Negl Trop Dis. 2026 Nov 10;20(11):e0014843. doi: 10.1371/journal.pntd.0014843 (PMC13766611; doi:10.1371/journal.pntd.0014843)
Supplement: Supplementary file 1 [file pntd.0014843.s001.docx]

**S1 Table.** Study-level matrix of predictor domains, modelling approaches, and validation practices in lymphatic filariasis risk prediction studies.

| **Study** | **Predictor domains** | | | | | **Predictor selection strategies** | | | **Modelling approaches** | | | | | | **Model validation strategies** | | | **Validation technique** | | | | **Model performance metrics** | | | | |
| --- | --- | --- | --- | --- | --- | --- | --- | --- | --- | --- | --- | --- | --- | --- | --- | --- | --- | --- | --- | --- | --- | --- | --- | --- | --- | --- |
|  | **Climatic** | **Environmental** | **Socio-demographic** | **Vector-related** | **MDA/Programmatic** | **Expert-driven inclusion** | **Univariate screening** | **Algorithm-based selection** | **Bayesian / Spatial** | **Conventional statistical** | **Machine learning** | **Ecological niche modelling** | **Hybrid / Ensemble** | **Other Models*** | **Internal validation** | **External validation** | **Partial / limited validation** | **Cross-validation** | **Split-sample validation** | **Retrospective validation** | **Performance metrics only** | **Discrimination (AUC / ROC)** | **Accuracy / Error metrics** | **Qualitative / Visual assessment** | **Calibration metrics** |  |
| (Palaniyandi, 2014) | 1 | 1 | 1 |  |  | 1 |  |  | 1 |  |  |  |  |  |  |  |  |  |  |  | 1 |  |  | 1 |  |  |
| (Slater and Michael, 2013) | 1 | 1 | 1 |  |  |  |  | 1 | 1 |  |  |  |  |  | 1 |  | 1 |  |  |  | 1 |  | 1 |  |  |  |
| (Lemin et al., 2022) | 1 | 1 | 1 |  |  |  | 1 |  |  | 1 |  |  |  |  |  |  |  |  |  |  | 1 |  | 1 |  |  |  |
| (Eneanya et al., 2023) | 1 | 1 | 1 |  |  |  |  | 1 |  |  | 1 |  |  |  | 1 |  |  | 1 |  |  |  |  | 1 |  |  |  |
| (Mayfield et al., 2020) | 1 | 1 |  |  |  |  |  | 1 |  |  |  |  | 1 |  | 1 |  |  |  |  | 1 |  |  | 1 | 1 |  |  |
| (Kwarteng et al., 2021) | 1 | 1 | 1 |  |  |  |  | 1 |  |  | 1 |  |  |  | 1 |  |  | 1 |  |  |  | 1 | 1 |  |  |  |
| (Eneanya et al., 2018) | 1 | 1 |  |  |  |  |  | 1 |  |  |  | 1 |  |  | 1 |  |  |  | 1 |  |  | 1 | 1 |  |  |  |
| (Slater and Michael, 2012) | 1 | 1 | 1 |  |  |  |  | 1 |  |  |  | 1 |  |  | 1 |  |  |  | 1 |  |  | 1 |  |  |  |  |
| (Kondeti et al., 2019) |  |  | 1 |  |  |  |  | 1 |  |  | 1 |  |  |  | 1 |  |  | 1 |  |  |  |  | 1 |  |  |  |
| (Eneanya et al., 2019) | 1 | 1 | 1 |  |  |  |  | 1 |  |  | 1 |  |  |  | 1 |  |  | 1 |  |  |  |  | 1 |  |  |  |
| (Sabesan et al., 2013) | 1 | 1 |  |  |  | 1 |  |  |  | 1 |  |  |  |  |  | 1 |  |  |  |  | 1 | 1 |  |  | 1 |  |
| (Moraga et al., 2015) | 1 | 1 | 1 | 1 |  |  |  | 1 | 1 |  |  |  |  |  | 1 |  |  | 1 |  |  |  |  | 1 |  |  |  |
| (Cadavid Restrepo et al., 2023) | 1 | 1 | 1 |  |  |  |  | 1 | 1 |  |  |  |  |  | 1 |  |  |  | 1 |  |  | 1 |  |  |  |  |
| (Sabesan et al., 2006) | 1 | 1 |  |  |  | 1 |  |  |  | 1 |  |  |  |  |  | 1 |  |  |  |  | 1 |  | 1 |  | 1 |  |
| (Stensgaard et al., 2011) | 1 | 1 | 1 |  |  |  |  | 1 | 1 |  |  |  |  |  | 1 |  |  |  | 1 |  |  |  | 1 |  |  |  |
| (Berg Soto et al., 2018) |  |  |  |  | 1 | 1 |  |  |  | 1 |  |  |  |  |  |  |  |  |  |  | 1 |  | 1 |  |  |  |
| (Barrett et al., 2024) | 1 | 1 | 1 |  |  | 1 |  |  | 1 |  |  |  | 1 |  |  |  |  |  |  |  | 1 |  | 1 |  |  |  |
| (Okunlola et al., 2025) |  | 1 |  | 1 |  | 1 |  |  | 1 |  |  |  |  |  |  |  |  |  |  |  | 1 |  | 1 |  |  |  |
| (Andrade-Pacheco et al., 2020) | 1 | 1 |  |  |  | 1 |  |  |  |  |  |  | 1 |  | 1 |  |  | 1 |  |  |  |  | 1 |  |  |  |
| (Amaliana et al., 2018) |  |  | 1 |  |  | 1 |  |  | 1 |  |  |  |  |  |  |  |  |  |  |  | 1 |  | 1 |  |  |  |
| (Mutahar et al., 2017) | 1 | 1 | 1 |  |  | 1 |  |  |  |  |  |  |  | 1 |  |  |  |  |  |  | 1 |  |  |  |  |  |
| (Hassan, 2004) | 1 | 1 |  |  |  |  |  | 1 |  |  |  |  |  | 1 |  |  | 1 |  |  |  | 1 |  | 1 | 1 |  |  |
| (Gyapong et al., 2002) |  |  |  |  |  |  |  |  | 1 |  |  |  |  |  |  | 1 |  |  |  |  | 1 |  |  | 1 |  |  |
| (Lindsay and Thomas, 2000) | 1 |  |  |  |  | 1 |  |  |  | 1 |  |  |  |  | 1 |  |  |  | 1 |  |  |  | 1 |  |  |  |
| (Sumboh et al., 2024) |  |  | 1 |  | 1 |  | 1 |  | 1 |  |  |  |  | 1 |  |  |  |  |  |  | 1 |  |  | 1 |  |  |
| (Schlüter et al., 2016) |  | 1 |  |  |  | 1 |  |  |  | 1 |  |  |  |  |  | 1 | 1 |  |  |  | 1 |  | 1 | 1 |  |  |

* Other Models: (Mutahar et al., 2017) - GIS-based weighted overlay scoring, (Hassan, 2004) - Descriptive interpolation, (Sumboh et al., 2024) - Ordinary Least Squares (OLS) regression, Getis-Ord Gi hotspot analysis, Anselin Local Moran's I (LISA), and Kernel Density Estimation (KDE).
