# Supplementary material for: Lymphatic filariasis risk prediction models: A systematic review of modelling approaches, predictors, validation practices, and methodological quality
Source: PLoS Negl Trop Dis. 2026 Nov 10;20(11):e0014843. doi: 10.1371/journal.pntd.0014843 (PMC13766611; doi:10.1371/journal.pntd.0014843)
Supplement: Supplementary file 2 [file pntd.0014843.s002.docx]

# S2 Table. Predictor domains used in lymphatic filariasis risk prediction models (n = 26)

| **Predictor categories** | **No. of Studies (%)*** | **Studies Reported** |
| --- | --- | --- |
| **Climatic factors (19, 73.1%)** | |  |
| Temperature | 18 (69.2%) | (Palaniyandi, 2014), (Slater and Michael, 2013), (Eneanya et al., 2023), (Mayfield et al., 2020), (Kwarteng et al., 2021), (Eneanya et al., 2018), (Slater and Michael, 2012), (Eneanya et al., 2019), (Sabesan et al., 2013), (Moraga et al., 2015), (Cadavid Restrepo et al., 2023), (Sabesan et al., 2006), (Stensgaard et al., 2011), (Barrett et al., 2024), (Andrade-Pacheco et al., 2020), (Mutahar et al., 2017), (Hassan, 2004), (Lindsay and Thomas, 2000) |
| Rainfall | 18 (69.2%) | (Palaniyandi, 2014), (Slater and Michael, 2013), (Lemin et al., 2022), (Eneanya et al., 2023), (Mayfield et al., 2020), (Kwarteng et al., 2021), (Eneanya et al., 2018), (Slater and Michael, 2012), (Eneanya et al., 2019), (Sabesan et al., 2013), (Moraga et al., 2015), (Cadavid Restrepo et al., 2023), (Sabesan et al., 2006), (Stensgaard et al., 2011), (Barrett et al., 2024), (Andrade-Pacheco et al., 2020), (Hassan, 2004), (Lindsay and Thomas, 2000) |
| Humidity | 6 (23.1%) | (Palaniyandi, 2014), (Sabesan et al., 2013), (Sabesan et al., 2006), (Barrett et al., 2024), (Mutahar et al., 2017), (Hassan, 2004) |
| Precipitation extremes / Seasonality | 4 (15.4%) | (Kwarteng et al., 2021), (Cadavid Restrepo et al., 2023), (Andrade-Pacheco et al., 2020), (Lindsay and Thomas, 2000) |
| **Environmental factors** **(20, 76.9%)** | |  |
| Vegetation indices (NDVI / EVI) | 9 (34.6%) | (Palaniyandi, 2014), (Slater and Michael, 2013), (Kwarteng et al., 2021), (Slater and Michael, 2012), (Eneanya et al., 2019), (Moraga et al., 2015), (Stensgaard et al., 2011), (Okunlola et al., 2025), (Hassan, 2004) |
| Altitude/topography | 17 (65.4%) | (Palaniyandi, 2014), (Slater and Michael, 2013), (Lemin et al., 2022), (Eneanya et al., 2023), (Mayfield et al., 2020), (Kwarteng et al., 2021), (Eneanya et al., 2018), (Slater and Michael, 2012), (Eneanya et al., 2019), (Sabesan et al., 2013), (Moraga et al., 2015), (Cadavid Restrepo et al., 2023), (Sabesan et al., 2006), (Stensgaard et al., 2011), (Barrett et al., 2024), (Andrade-Pacheco et al., 2020), (Mutahar et al., 2017) |
| Land cover/land use | 8 (30.8%) | (Palaniyandi, 2014), (Lemin et al., 2022), (Kwarteng et al., 2021), (Eneanya et al., 2018), (Eneanya et al., 2019), (Moraga et al., 2015), (Cadavid Restrepo et al., 2023), (Hassan, 2004) |
| Distance to water / wetness / soil indices | 8 (30.8%) | (Mayfield et al., 2020), (Kwarteng et al., 2021), (Eneanya et al., 2018), (Eneanya et al., 2019), (Cadavid Restrepo et al., 2023), (Stensgaard et al., 2011), (Andrade-Pacheco et al., 2020), (Hassan, 2004) |
| Night-time lights / built environment proxies | 4 (15.4%) | (Mayfield et al., 2020), (Kwarteng et al., 2021), (Eneanya et al., 2018), (Eneanya et al., 2019) |
| **Entomological (vector) factors (2, 7.7%)** | |  |
| Vector density / vector distribution indices | 2 (7.7%) | (Moraga et al., 2015), (Hassan, 2004) |
| **Socio-demographic factors** **(15, 57.7%)** | |  |
| Population density | 10 (38.5%) | (Palaniyandi, 2014), (Slater and Michael, 2013), (Lemin et al., 2022), (Kwarteng et al., 2021), (Slater and Michael, 2012), (Moraga et al., 2015), (Cadavid Restrepo et al., 2023), (Barrett et al., 2024), (Mutahar et al., 2017), (Sumboh et al., 2024) |
| Socioeconomic proxies (housing, sanitation, education, MDA participation) | 9 (34.6%) | (Eneanya et al., 2023), (Kwarteng et al., 2021), (Kondeti et al., 2019), (Cadavid Restrepo et al., 2023), (Stensgaard et al., 2011), (Berg Soto et al., 2018), (Okunlola et al., 2025), (Amaliana et al., 2018)**,** (Sumboh et al., 2024) |

* Percentages reflect proportion of studies including at least one variable in the domain.
