# Supplementary material for: Lymphatic filariasis risk prediction models: A systematic review of modelling approaches, predictors, validation practices, and methodological quality
Source: PLoS Negl Trop Dis. 2026 Nov 10;20(11):e0014843. doi: 10.1371/journal.pntd.0014843 (PMC13766611; doi:10.1371/journal.pntd.0014843)
Supplement: Supplementary file 3 [file pntd.0014843.s003.docx]

**S3 Table.** Study-level PROBAST risk of bias assessment for included lymphatic filariasis risk prediction studies.

| **Study (Author, Year)** | **Participants** | **Predictors** | **Outcome** | **Analysis** | **Overall risk of bias** |
| --- | --- | --- | --- | --- | --- |
| (Palaniyandi, 2014) | Low | High | Low | High | High |
| (Slater and Michael, 2013) | Low | Low | Low | High | High |
| (Lemin et al., 2022) | Low | Low | Low | High | High |
| (Eneanya et al., 2023) | Low | Low | Low | Unclear | High |
| (Mayfield et al., 2020) | Low | Low | Low | Unclear | High |
| (Kwarteng et al., 2021) | Low | Low | Low | Unclear | High |
| (Eneanya et al., 2018) | Low | Low | Low | High | High |
| (Slater and Michael, 2012) | Low | Low | Low | High | High |
| (Kondeti et al., 2019) | Low | Low | Low | Unclear | High |
| (Eneanya et al., 2019) | Low | Low | Low | Unclear | High |
| (Sabesan et al., 2013) | Low | Low | Low | Unclear | High |
| (Moraga et al., 2015) | Low | Low | Low | Unclear | High |
| (Cadavid Restrepo et al., 2023) | Low | Low | Low | Unclear | High |
| (Sabesan et al., 2006) | Low | Low | Low | Unclear | High |
| (Stensgaard et al., 2011) | Low | Low | Low | Unclear | High |
| (Berg Soto et al., 2018) | Low | High | Low | High | High |
| (Barrett et al., 2024) | Low | Low | Low | High | High |
| (Okunlola et al., 2025) | Low | Low | Low | High | High |
| (Andrade-Pacheco et al., 2020) | Low | Low | Low | Unclear | High |
| (Amaliana et al., 2018) | Low | Low | Low | High | High |
| (Mutahar et al., 2017) | Low | High | Low | High | High |
| (Hassan, 2004) | High | High | Low | Unclear | High |
| (Gyapong et al., 2002) | High | High | Low | Unclear | High |
| (Lindsay and Thomas, 2000) | Low | Low | Low | Unclear | High |
| (Sumboh et al., 2024) | Low | Low | Low | High | High |
| (Schlüter et al., 2016) | Low | Low | Low | Unclear | High |

**Note:**

- Participants domain was judged as high risk when studies relied on highly aggregated or poorly defined populations without clear sampling frameworks.
- Predictors domain was judged as high risk when predictor measurement, timing, or selection procedures were insufficiently reported.
- Outcome domain was judged as low risk when outcomes were clearly defined and measured using established diagnostic methods.
- Analysis domain was judged as high risk when studies reported limited or no validation, unclear handling of overfitting, or absence of calibration assessment.
- Overall risk of bias was classified as high when at least one PROBAST domain was judged as high or unclear risk, consistent with the decision rule specified in the Methods.
