# Supplementary material for: Lymphatic filariasis risk prediction models: A systematic review of modelling approaches, predictors, validation practices, and methodological quality
Source: PLoS Negl Trop Dis. 2026 Nov 10;20(11):e0014843. doi: 10.1371/journal.pntd.0014843 (PMC13766611; doi:10.1371/journal.pntd.0014843)
Supplement: Supplementary file 4 [file pntd.0014843.s004.docx]

**S4 Table.** Study-level outcome and methodological characteristics

| **Study** | **Outcome type** | **Modelling approach** | **Predictor domains** | **Validation strategy** | **Validation technique** | **Performance assessment** | **External validation characteristics** |
| --- | --- | --- | --- | --- | --- | --- | --- |
| (Palaniyandi, 2014) | Composite risk/vulnerability Classification | Bayesian/spatial | Climatic; Environmental; Socio-demographic |  | Performance metrics only | Qualitative/visual | Not applicable |
| (Slater and Michael, 2013) | Infection Prevalence (continuous) | Bayesian/spatial | Climatic; Environmental; Socio-demographic | Internal; Partial/limited | Performance metrics only | Accuracy/error | Not applicable |
| (Lemin et al., 2022) | Infection Prevalence (continuous) | Conventional statistical | Climatic; Environmental; Socio-demographic |  | Performance metrics only | Accuracy/error | Not applicable |
| (Eneanya et al., 2023) | Infection Prevalence (continuous) | Machine learning | Climatic; Environmental; Socio-demographic | Internal | Cross-validation | Accuracy/error | Not applicable |
| (Mayfield et al., 2020) | Infection Prevalence (continuous) | Hybrid/ensemble | Climatic; Environmental | Internal | Retrospective | Accuracy/error; Qualitative/visual | Not applicable |
| (Kwarteng et al., 2021) | Binary infection/endemicity status | Machine learning | Climatic; Environmental; Socio-demographic | Internal | Cross-validation | Discrimination/AUC; Accuracy/error | Not applicable |
| (Eneanya et al., 2018) | Environmental/vector suitability | ENM | Climatic; Environmental | Internal | Split-sample | Discrimination/AUC; Accuracy/error | Not applicable |
| (Slater and Michael, 2012) | Environmental/vector suitability | ENM | Climatic; Environmental; Socio-demographic | Internal | Split-sample | Discrimination/AUC | Not applicable |
| (Kondeti et al., 2019) | Binary infection/endemicity status | Machine learning | Socio-demographic | Internal | Cross-validation | Accuracy/error | Not applicable |
| (Eneanya et al., 2019) | Infection Prevalence (continuous) | Machine learning | Climatic; Environmental; Socio-demographic | Internal | Cross-validation | Accuracy/error | Not applicable |
| (Sabesan et al., 2013) | Binary infection/endemicity status | Conventional statistical | Climatic; Environmental | External | Performance metrics only | Discrimination/AUC; Calibration | 125 previously unsurveyed districts, national scale |
| (Moraga et al., 2015) | Infection Prevalence (continuous) | Bayesian/spatial | Climatic; Environmental; Socio-demographic; Vector-related | Internal | Cross-validation | Accuracy/error | Not applicable |
| (Cadavid Restrepo et al., 2023) | Binary infection/endemicity status | Bayesian/spatial | Climatic; Environmental; Socio-demographic | Internal | Split-sample | Discrimination/AUC | Not applicable |
| (Sabesan et al., 2006) | Binary infection/endemicity status | Conventional statistical | Climatic; Environmental | External | Performance metrics only | Accuracy/error; Calibration | 60 new geo-coded sites, not used in development, same region |
| (Stensgaard et al., 2011) | Infection Prevalence (continuous) | Bayesian/spatial | Climatic; Environmental; Socio-demographic | Internal | Split-sample | Accuracy/error | Not applicable |
| (Berg Soto et al., 2018) | Infection Prevalence (continuous) | Conventional statistical | MDA/programmatic |  | Performance metrics only | Accuracy/error | Not applicable |
| (Barrett et al., 2024) | Case burden/counts | Bayesian/spatial; Hybrid/ensemble | Climatic; Environmental; Socio-demographic |  | Performance metrics only | Accuracy/error | Not applicable |
| (Okunlola et al., 2025) | Infection Prevalence (continuous) | Bayesian/spatial; | Environmental; Vector-related |  | Performance metrics only | Accuracy/error | Not applicable |
| (Andrade-Pacheco et al., 2020) | Composite risk/vulnerability Classification | Hybrid/ensemble | Climatic; Environmental | Internal | Cross-validation | Accuracy/error | Not applicable |
| (Amaliana et al., 2018) | Case burden/counts | Bayesian/spatial | Socio-demographic |  | Performance metrics only | Accuracy/error | Not applicable |
| (Mutahar et al., 2017) | Composite risk/vulnerability Classification | Other models | Climatic; Environmental; Socio-demographic |  | Performance metrics only |  | Not applicable |
| (Hassan, 2004) | Composite risk/vulnerability Classification | Other models | Climatic; Environmental | Partial/limited | Performance metrics only | Accuracy/error; Qualitative/visual | Not applicable |
| (Gyapong et al., 2002) | Infection Prevalence (continuous) | Bayesian/spatial |  | External | Performance metrics only | Qualitative/visual | independent re-survey data |
| (Lindsay and Thomas, 2000) | Binary infection/endemicity status | Conventional statistical | Climatic | Internal | Split-sample | Accuracy/error | Not applicable |
| (Sumboh et al., 2024) | Composite risk/vulnerability Classification | Bayesian/spatial; Other models | Socio-demographic; MDA/programmatic |  | Performance metrics only | Qualitative/visual | Not applicable |
| (Schlüter et al., 2016) | Infection Prevalence (continuous) | Conventional statistical | Environmental | External; Partial/limited | Performance metrics only | Accuracy/error; Qualitative/visual; | 253 new villages in Cameroon, Gabon and Equatorial Guinea, not used in development |
