# Supplementary material for: Lymphatic filariasis risk prediction models: A systematic review of modelling approaches, predictors, validation practices, and methodological quality
Source: PLoS Negl Trop Dis. 2026 Nov 10;20(11):e0014843. doi: 10.1371/journal.pntd.0014843 (PMC13766611; doi:10.1371/journal.pntd.0014843)
Supplement: Supplementary file 5 [file pntd.0014843.s005.docx]

**S5 Table.** Study-level assessment of temporal alignment between predictor and outcome data.

| **Temporal category** | **Studies** | **No. of studies (%)** |
| --- | --- | --- |
| Explicitly contemporaneous/matched | (Lemin et al., 2022), (Mayfield et al., 2020), (Kondeti et al., 2019), (Cadavid Restrepo et al., 2023), (Stensgaard et al., 2011), (Berg Soto et al., 2018), (Okunlola et al., 2025), (Amaliana et al., 2018), (Hassan, 2004), (Gyapong et al., 2002), (Sumboh et al., 2024), (Schlüter et al., 2016) | 12/26 (46.2%) |
| Long-term averages / mismatch likely | (Slater and Michael, 2013), (Eneanya et al., 2018), (Slater and Michael, 2012), (Sabesan et al., 2013), (Sabesan et al., 2006), (Andrade-Pacheco et al., 2020), (Lindsay and Thomas, 2000) | 7/26 (26.9%) |
| Partial/attempted matching | (Eneanya et al., 2023), (Eneanya et al., 2019) | 2/26 (7.7%) |
| Not reported | (Palaniyandi, 2014), (Kwarteng et al., 2021), (Barrett et al., 2024), (Mutahar et al., 2017) | 4/26 (15.4%) |
| Mismatch explicitly acknowledged | (Moraga et al., 2015) | 1/26 (3.8%) |
