# Supplementary material for: Lymphatic filariasis risk prediction models: A systematic review of modelling approaches, predictors, validation practices, and methodological quality
Source: PLoS Negl Trop Dis. 2026 Nov 10;20(11):e0014843. doi: 10.1371/journal.pntd.0014843 (PMC13766611; doi:10.1371/journal.pntd.0014843)
Supplement: Supplementary file 6 [file pntd.0014843.s008.docx]

**Search Strategy**

**Scopus:**

**LF:**

TITLE-ABS-KEY(("lymphatic filariasis" OR filariasis OR "lymphatic filariasis elimination" OR "Wuchereria bancrofti" OR Brugia OR "Brugia malayi" OR "Brugia timori") AND (model* OR predict* OR forecast* OR "risk model*" OR "risk prediction" OR "spatial model*" OR "geostatistical model*" OR "time series" OR "early warning system*" OR "transmission model*" OR "ecological niche model*" OR MaxEnt OR "machine learning" OR "deep learning" OR "artificial intelligence" OR Bayesian))

**Web of Science:**

**LF:**

TS=(("lymphatic filariasis" OR filariasis OR "Wuchereria bancrofti" OR "Brugia malayi" OR "Brugia timori" OR Brugia) AND (model* OR predict* OR forecast* OR "risk model*" OR "risk prediction" OR "spatial model*" OR "geostatistical model*" OR "time series" OR "early warning system*" OR "transmission model*" OR "ecological niche model*" OR MaxEnt OR "machine learning" OR "deep learning" OR "artificial intelligence" OR Bayesian))

**PubMed:**

**LF:**

(("lymphatic filariasis"[Title/Abstract] OR filariasis[Title/Abstract] OR "Wuchereria bancrofti"[Title/Abstract] OR "Brugia malayi"[Title/Abstract] OR "Brugia timori"[Title/Abstract] OR Brugia[Title/Abstract]) AND (model*[Title/Abstract] OR predict*[Title/Abstract] OR forecast*[Title/Abstract] OR "risk model*"[Title/Abstract] OR "risk prediction"[Title/Abstract] OR "spatial model*"[Title/Abstract] OR "geostatistical model*"[Title/Abstract] OR "time series"[Title/Abstract] OR "early warning system*"[Title/Abstract] OR "transmission model*"[Title/Abstract] OR "ecological niche model*"[Title/Abstract] OR MaxEnt[Title/Abstract] OR "machine learning"[Title/Abstract] OR "deep learning"[Title/Abstract] OR "artificial intelligence"[Title/Abstract] OR Bayesian[Title/Abstract]))

**Google Scholar**

**LF:**

("lymphatic filariasis" OR filariasis OR "lymphatic filariasis elimination" OR "Wuchereria bancrofti" OR Brugia OR "Brugia malayi" OR "Brugia timori") AND (model* OR predict* OR forecast* OR "risk model*" OR "risk prediction" OR "spatial model*" OR "geostatistical model*" OR "time series" OR "early warning system*" OR "transmission model*" OR "ecological niche model*" OR MaxEnt OR "machine learning" OR "deep learning" OR "artificial intelligence" OR Bayesian)
