# Supplementary material for: Lymphatic filariasis risk prediction models: A systematic review of modelling approaches, predictors, validation practices, and methodological quality
Source: PLoS Negl Trop Dis. 2026 Nov 10;20(11):e0014843. doi: 10.1371/journal.pntd.0014843 (PMC13766611; doi:10.1371/journal.pntd.0014843)
Supplement: Supplementary file 7 [file pntd.0014843.s010.docx]

| **S.No** | **Classification of Framework** | **Definition** | **Qualified Variables** |
| --- | --- | --- | --- |
| **1** | Climatic Predictors | Temperature, rainfall/precipitation, humidity, relative/absolute humidity, seasonal climate indices. | Mean/min/max temperature; rainfall totals, intensity, lags; relative or absolute humidity; humidity saturation deficit; seasonal climate indices derived from meteorological records. |
| **2** | Environmental Predictors | Ecological, geographic, or land-surface variables reflecting the physical landscape. | NDVI/EVI; land use/land cover (LULC); elevation/altitude/slope; distance to water bodies; wetness index; soil type/texture; night-time lights used as an environmental proxy. |
| **3** | Entomological Factors | Actual entomological/vector survey data used as model input, or a vector suitability layer derived within this study. | Anopheles/Culex/Mansonia density; distribution survey data; breeding habitat surveys; vector species distribution records used directly; suitability layer re-derived within this study. |
| 4 | MDA/Programmatic Data Integration | MDA exposure data is the analogous and programmatically critical variable for LF. | MDA history/coverage/exposure used as a predictor in the model; treatment-round data incorporated; MDA presence/absence as a model covariate. |
| **5** | Socio-demographic Predictors | Human population or social-structural variables describing people, not landscape. | Population density; age/sex structure; housing quality (drainage, latrines, roof type); income/occupation; MDA participation/history; awareness/KAP variables; healthcare access. |
| **6** | Expert-Driven Inclusion | Authors explicitly state that predictors were chosen based on prior epidemiological knowledge, domain expertise, or published literature — a theory-first decision made before examining the data. | “Variables were selected based on their known association with LF transmission”; guided by epidemiological theory; literature review used to compile candidate list before data analysis. |
| **7** | Univariate Screening | Individual statistical tests applied to each predictor separately against the outcome, with results used to filter variables before multivariable modelling. | Univariate logistic/Poisson regression with stated threshold (e.g. p<0.20); Pearson/Spearman correlation used to filter predictors; chi-square or Fisher’s exact test used as inclusion filter; Mann-Whitney U tests used to screen predictors; bivariate analysis to shortlist candidates. |
| **8** | Algorithm-Based Selection | A computational or mathematical algorithm applied to rank, shrink, or eliminate predictors. | Stepwise regression (AIC/BIC, forward/backward); VIF-based exclusion used as a selection step; RF variable importance used explicitly to filter before a DIFFERENT final model; PCA for dimensionality reduction; gain-ratio/boosted-tree-based variable importance used as a filter. |
| **9** | Bayesian / Spatial | Study employs a Bayesian statistical framework or a spatially structured model that explicitly accounts for spatial autocorrelation or spatial random effects. | Bayesian hierarchical models; BYM/BYM2; INLA/INLA-SPDE; CAR/ICAR; MCMC (OpenBUGS, WinBUGS, Stan, JAGS); Bayesian geostatistics; kriging used as a predictive model; GWR; spatial scan statistics producing risk estimates; PrevMap/geostatistical binomial models. |
| **10** | Conventional Statistical | Standard frequentist regression or time-series models without machine learning or Bayesian spatial components. | Logistic regression; Poisson/negative binomial regression; multivariate linear regression; GLM; GAM (smooth GLM extensions — NOT ML); stepwise multivariate regression; non-linear regression (exponential/power functions) used as predictive models. |
| **11** | Machine Learning | Data-driven algorithms that learn patterns from training data without requiring explicit parametric model specification. | Random Forest (RF); Quantile Regression Forest (QRF); GBM/boosted trees used as a classifier; ANN; decision tree classifiers (NB, J48, JRip, LMT, CART, PNN); KNN; SVM; WEKA-implemented classifiers. |
| **12** | Ecological Niche Modelling (ENM) | Models that relate occurrence or presence data to environmental conditions to estimate habitat suitability or geographic risk distribution. | Maxent (applied by this study); GARP; BIOCLIM; BIOMD2/BIOMOD ensemble SDMs; habitat suitability modelling with presence-only or presence-background data. |
| **13** | Hybrid / Ensemble | A single integrated model or prediction that formally combines outputs from two or more distinct modelling approaches in a stated multi-stage pipeline. | RF for environmental signal feeding into a geostatistical model; ensemble of GBM+RF within BIOMOD; adaptive random forest + Bayesian geostatistical spatial model (two-stage pipeline where output of model A feeds model B). |
| **14** | Other Models | Modelling frameworks that do not fit categories 3.1–3.5. | GIS-based overlay/scoring without statistical fitting; kernel density/Anselin Local Moran’s I/Getis-Ord hotspot analysis used as the primary modelling output; discriminant analysis used as primary classification model. |
| **15** | Internal Validation | Validation performed using data from the SAME study dataset, population, and geographic region. | Any technique in 4.1–4.3 applied within the study’s own data; train/test split from the same cohort; bootstrap resampling on the same data. |
| **16** | External Validation | Validation uses a COMPLETELY INDEPENDENT dataset: different geographic area, different population, or a separate cohort not part of model development. | Model trained in Region A, validated in Region B using that region’s independent data; model validated on a prospective dataset collected after model development in a different setting. |
| **17** | Partial / Limited Validation | Some validation is attempted but incomplete, informal, or insufficient to fully characterise generalisation. | Visual comparison of observed vs predicted without quantitative metrics; validation on <10% of records; “face validity” expert judgement; sensitivity analysis used as validation proxy; entomological/epidemiological comparison between communities without formal external validation. |
| **18** | Cross-validation | Dataset systematically partitioned into multiple folds for iterative training and testing — the model is trained K-1 times, each time tested on the held-out fold. | K-fold CV (5-fold, 10-fold, etc.); LOOCV; leave-one-region/year-out; repeated k-fold CV; spatial cross-validation. |
| **19** | Split-sample Validation | Data divided ONCE into a training set and a separate test/holdout set. | Single train/test partition (70/30, 75/25, 80/20); holdout set defined once; training/test data using a single random or stratified partition |
| **20** | Performance Metrics Only | No formal data-splitting or resampling validation. Only in-sample fit statistics reported. | DIC/WAIC for model comparison (no data splitting); AIC/BIC for model selection; R² or deviance from training data only; sensitivity/specificity on full training dataset without holdout. |
| **21** | Retrospective Validation | This approach assesses the model's temporal generalizability using an explicit validation design, rather than a simple random or continuous split of the same dataset. | Model trained on data from one period, applied to predict/validate against a genuinely distinct later (or earlier) time period within the SAME population/region, explicitly framed as the validation design. |
| **22** | Discrimination (AUC/ROC) | The Area Under the ROC Curve (AUC) or the ROC curve itself is explicitly computed and reported for this study's predictive model. | AUC value explicitly stated; ROC curve plotted; C-statistic; partial AUC; AUROC computed on test or validation data. |
| **23** | Accuracy / Error Metrics | Quantitative error or fit statistics reported for this study's own model. | RMSE, MAE, R², Pearson r, sensitivity/specificity/PPV/NPV, accuracy %, DIC, WAIC (Bayesian predictive fit — codes here, NOT calibration), AIC/BIC for variant comparison, F1-score, precision, recall. |
| **24** | Qualitative / Visual Assessment | Spatial risk maps, hotspot maps, or visual comparisons produced as model output and used for evaluation. | Risk maps/predicted-vs-observed maps generated by the model and presented as a result used for evaluation; spatial pattern comparison without a formal statistic; time-series plot of observed vs predicted used to assess model fit. |
| **25** | Calibration Metrics | Metrics that specifically compare EXPECTED vs. OBSERVED outcome probabilities or event frequencies — assessing whether predicted probabilities agree with observed rates. | Hosmer-Lemeshow goodness-of-fit test; Brier score; calibration plot (observed proportions vs predicted probabilities across risk groups); E/O ratio; calibration slope and intercept; reliability diagram. |
| **26** | Temperature | Any measure of air temperature used directly as a model predictor. | mean, min, max, diurnal range, anomaly |
| **27** | Rainfall / Precipitation | Any measure of precipitation used directly as a model predictor. | total, intensity, lags, anomaly |
| **28** | Humidity | Relative humidity, absolute humidity, specific humidity, or dew point used as a predictor. | relative, absolute, saturation deficit, vapour pressure |
| **29** | Seasonal Indicators / Climate Indices | Derived composite climate variables or categorical indicators of seasonal patterns used as predictors. | monsoon indicator, ENSO, SPEI, monthly climate summaries |
| **30** | Vegetation Indices (NDVI/EVI) | Satellite-derived vegetation index used as a predictor. | NDVI, EVI, SAVI |
| **31** | Altitude / Elevation / Topography | Digital elevation model (DEM) data or topographic derivatives used as predictors. | slope, aspect, DEM |
| **32** | Land Use / Land Cover (LULC) | Land classification data (urban, agricultural, forest, water) used as a predictor. | LULC — urban/agricultural/forest fraction, impervious surface, deforestation index |
| **33** | Distance to Water Bodies / Wetness / Soil indices | Proximity to surface water or hydrological wetness measures used as predictors. | distance to rivers/lakes, TWI, flood plain, JRC surface water, soil type/texture, soil drainage class. |
| **34** | Night-time Lights / Built environmental proxies | Any ecological or landscape variable not captured in the above four rows. | Night-time lights as environmental proxy, distance to roads as habitat connectivity metric |
| **35** | Vector density / Vector distribution indices | presence records and density of vectors used directly as predictors | Anopheles/Culex/Mansonia |
| **36** | Breeding Indices | The study uses breeding habitat indices (larval density, breeding site presence), mosquito infection rates (proportion of vectors infected with Wuchereria bancrofti), or similar vector infection or productivity indices as model predictors. | Kondeti 2019 — "breeding habitats near home" is a household-level predictor variable capturing presence of mosquito breeding sites near the respondent's house |
| **37** | Population Density | The number of persons per unit area used as a predictor. | persons per km², WorldPop/LandScan/GPW gridded density |
| **38** | Housing and Sanitation (Socioeconomic proxies) | Variables capturing physical housing conditions or water/sanitation infrastructure, education, MDA participation. | Drainage systems, latrine coverage, roof type, water storage practices, healthy housing indicators) — LF-critical domain given mosquito-breeding/housing-quality links. |
| **39** | Infection Prevalence (continuous) | Model predicts mf or antigenemia prevalence as a continuous measure | Predicted community mf prevalence %; antigenemia prevalence surface; Bayesian geostatistical prevalence estimates |
| **40** | Binary Infection / Endemicity Status | Model predicts presence/absence of infection, or endemic vs non-endemic classification (binary outcome) | LF presence/absence at survey site; endemic status per district; endemicity threshold classification |
| **41** | Environmental / Vector Suitability | Continuous suitability/occurrence-probability index from ENM using presence-only/presence-background data; NOT a human infection outcome | Maxent suitability for Anopheles/Culex vector; environmental suitability for LF transmission; GARP suitability map |
| **42** | Composite Risk / Vulnerability Classification | Categorical or index-based risk stratification (hotspot, vulnerability tier, endemicity tier) NOT derived from a fitted prevalence/incidence model | GIS overlay vulnerability index; hotspot classification via Getis-Ord / Local Moran’s I; GIS-based risk scoring |
| **43** | Case Burden / Counts | Absolute clinical case counts or burden estimates (not a rate or prevalence proportion) | Predicted LF clinical case counts per district/TA; Bayesian Poisson model for case burden estimation |
| **44** | National | Study area is an entire single country, regardless of how fine-grained the predictions are within that country | Palaniyandi 2014 (India national), Sabesan 2013 (India national) |
| **45** | Sub-national (Province / State / Region) | Study area is a defined sub-national administrative region — one or several first-level administrative divisions — not the whole country | Lemin 2022 / Cadavid Restrepo 2023 (American Samoa — a US territory comprising specific islands |
| **46** | District / Local Government Area | Study area is one or a few second-level administrative units | Kondeti 2019 (Karimnagar district, Telangana, India), Mutahar 2016 (Banyuasin District, South Sumatra, Indonesia) |
| **47** | Community / Village / Household | Study area is at community, village, or household spatial granularity within a small localised area | Sumboh 2024 (four rural communities in Ahanta West and Ellembelle districts, Ghana). |
| **48** | Multi-country / Regional | Study area deliberately spans two or more countries as a single, unified analytical unit. The model is fitted to or makes predictions across multiple countries simultaneously | Slater & Michael 2012 (continental Africa Maxent), Slater & Michael 2013 (continental Africa Bayesian GLSM) |
| **58** | Uncertainty Quantification | The model provides a quantitative measure of uncertainty around its predictions or parameter estimates. | 95% credible intervals; 95% CI on predicted values; prediction intervals; standard errors on spatial random effects; posterior distributions summarised; bootstrap CIs; Monte Carlo uncertainty bounds; ensemble spread as uncertainty measure. |
| **59** | Formal Calibration Plot Assessment | A calibration plot or equivalent visual comparison of observed vs. predicted probabilities is produced and reported for THIS study's model. | Hosmer-Lemeshow test; Brier score; calibration plot; reliability diagram; E/O ratio; calibration slope and intercept. |
| **60** | Missing Data Handling | The study explicitly describes how missing data in predictor or outcome variables were handled. | Imputation method described (MI, MICE, single imputation); complete-case analysis explicitly stated with justification; % missing per variable reported; model-based handling described (e.g. INLA handling); sensitivity analysis comparing imputed vs complete-case results. |
| **61** | Overfitting Control | The study employs explicit measures to prevent or detect model overfitting | LASSO/Ridge/Elastic Net penalties; regularisation multiplier in Maxent; CV explicitly framed as overfitting check; penalised likelihood; shrinkage estimators; EPV criterion explicitly discussed; train-vs-test performance comparison reported separately. |
| **62** | Operationalized Model | The model has been deployed or implemented in a form that can be actively used for public health decision-making beyond the research publication. | A working web-based risk dashboard or platform described in the paper; model integrated into a GIS tool used by health authorities; automated prediction pipeline deployed and actively running; model outputs used in real-time by health ministries. |
| **63** | Early Warning System | The model is described or designed as an early warning system for LF, with prospective or near-real-time risk signalling | Model explicitly described as an early warning system; generates prospective risk alerts for a defined surveillance area; near-real-time data feeds into model; model integrated into a syndromic surveillance platform with alerting functionality. |
| **64** | Government / Policy Integration | The model outputs are formally integrated with government health authorities, policy frameworks, or public health programmes | Explicit statement of formal collaboration with NTD programme/Ministry of Health; model outputs cited in official programme documentation; model results used in official elimination-programme decision-making; funding from government health agency with explicit policy translation mandate described. |

**PROBAST**

| **Domain** | **Assessment Criteria** |
| --- | --- |
| **Participants (D1)** | Studies using national surveillance datasets were classified as low risk. Studies relying on occurrence records, hospital-based data, or insufficiently described populations were classified as unclear risk. |
| **Predictors (D2)** | Predictor domains were considered low risk when climate variables were measured prior to outcome occurrence. Contemporaneous vector indices and ecological niche modelling (presence-only) predictors were classified as unclear risk. |
| **Outcome (D3)** | Models predicting LF incidence or binary LF risk with a clearly stated case definition were classified as low risk. Habitat suitability and spatial risk index outcomes were classified as unclear risk. |
| **Analysis**  **(D4)** | Low risk required formal validation, overfitting control, and uncertainty quantification. Studies reporting validation without adequate overfitting control or uncertainty assessment were classified as high risk. Studies reporting no validation were also classified as high risk. |
