# Supplementary material for: Lymphatic filariasis risk prediction models: A systematic review of modelling approaches, predictors, validation practices, and methodological quality
Source: PLoS Negl Trop Dis. 2026 Nov 10;20(11):e0014843. doi: 10.1371/journal.pntd.0014843 (PMC13766611; doi:10.1371/journal.pntd.0014843)
Supplement: Supplementary file 8 [file pntd.0014843.s012.docx]

| **Section and Topic** | **Item #** | **Checklist item** | **Location where item is reported** |
| --- | --- | --- | --- |
| **TITLE** | | |  |
| Title | 1 | Identify the report as a systematic review. | In Title Page (Page 1): Lymphatic filariasis risk prediction models: a systematic review of modelling approaches, predictors, validation practices, and methodological quality |
| **ABSTRACT** | | |  |
| Abstract | 2 | See the PRISMA 2020 for Abstracts checklist. | Abstract section (Page 4) |
| **INTRODUCTION** | | |  |
| Rationale | 3 | Describe the rationale for the review in the context of existing knowledge. | Introduction, paragraphs 1–6 (Pages 6-9) |
| Objectives | 4 | Provide an explicit statement of the objective(s) or question(s) the review addresses. | Final paragraph of Introduction: objectives listed (Page 8-9) |
| **METHODS** | | |  |
| Eligibility criteria | 5 | Specify the inclusion and exclusion criteria for the review and how studies were grouped for the syntheses. | Section 2.3 (Inclusion criteria; Exclusion criteria; Population and outcomes) (Page 11) |
| Information sources | 6 | Specify all databases, registers, websites, organisations, reference lists and other sources searched or consulted to identify studies. Specify the date when each source was last searched or consulted. | Section 2.2 Search Strategy (Pages 9-10) |
| Search strategy | 7 | Present the full search strategies for all databases, registers and websites, including any filters and limits used. | Section 2.2 and  Supplementary Material 1 (full search strings) (Pages 9-10) |
| Selection process | 8 | Specify the methods used to decide whether a study met the inclusion criteria of the review, including how many reviewers screened each record and each report retrieved, whether they worked independently, and if applicable, details of automation tools used in the process. | Section 2.3 & Figure 1. (Page 11) |
| Data collection process | 9 | Specify the methods used to collect data from reports, including how many reviewers collected data from each report, whether they worked independently, any processes for obtaining or confirming data from study investigators, and if applicable, details of automation tools used in the process. | Methods Section 2.4 (Page 12) and Supplementary Material 2. |
| Data items | 10a | List and define all outcomes for which data were sought. Specify whether all results that were compatible with each outcome domain in each study were sought (e.g. for all measures, time points, analyses), and if not, the methods used to decide which results to collect. | Methods Section 2.3.3 (Page 12) and Results 3.5. (Pages 15-16) |
|  | 10b | List and define all other variables for which data were sought (e.g. participant and intervention characteristics, funding sources). Describe any assumptions made about any missing or unclear information. | Methods Section 2.4 (Pages 12) and Tables 1-4 and Supplementary Table S1) |
| Study risk of bias assessment | 11 | Specify the methods used to assess risk of bias in the included studies, including details of the tool(s) used, how many reviewers assessed each study and whether they worked independently, and if applicable, details of automation tools used in the process. | Methods Section 2.5 (Page 12) |
| Effect measures | 12 | Specify for each outcome the effect measure(s) (e.g. risk ratio, mean difference) used in the synthesis or presentation of results. | Not applicable – no pooled quantitative effect estimates were calculated due to methodological heterogeneity. |
| Synthesis methods | 13a | Describe the processes used to decide which studies were eligible for each synthesis (e.g. tabulating the study intervention characteristics and comparing against the planned groups for each synthesis (item #5)). | Methods Section 2.6 (Page 13) and Table 1-4 and Supplementary Tables S1) |
|  | 13b | Describe any methods required to prepare the data for presentation or synthesis, such as handling of missing summary statistics, or data conversions. | Methods Section 2.6 (Page 13) |
|  | 13c | Describe any methods used to tabulate or visually display results of individual studies and syntheses. | Tables 1–5 Figures 1–4 Supplementary Figures 1-2, Supplementary Tables S1-S3 |
|  | 13d | Describe any methods used to synthesize results and provide a rationale for the choice(s). If meta-analysis was performed, describe the model(s), method(s) to identify the presence and extent of statistical heterogeneity, and software package(s) used. | Methods Section 2.6 (Page 13) |
|  | 13e | Describe any methods used to explore possible causes of heterogeneity among study results (e.g. subgroup analysis, meta-regression). | Section 3.7 (Pages 16-17), Figure 3 and Supplementary Figure S2. |
|  | 13f | Describe any sensitivity analyses conducted to assess robustness of the synthesized results. | Not applicable - Sensitivity analyses were not conducted due to absence of quantitative synthesis |
| Reporting bias assessment | 14 | Describe any methods used to assess risk of bias due to missing results in a synthesis (arising from reporting biases). | Assessment of reporting bias was not performed due to absence of pooled effect estimates. |
| Certainty assessment | 15 | Describe any methods used to assess certainty (or confidence) in the body of evidence for an outcome. | Not assessed due to absence of pooled quantitative effect estimates and high methodological heterogeneity. |
| **RESULTS** | | |  |
| Study selection | 16a | Describe the results of the search and selection process, from the number of records identified in the search to the number of studies included in the review, ideally using a flow diagram. | Section 3.1 |
|  | 16b | Cite studies that might appear to meet the inclusion criteria, but which were excluded, and explain why they were excluded. | Figure 1 (PRISMA flow diagram) and section 3.1 (Page 13) |
| Study characteristics | 17 | Cite each included study and present its characteristics. | Section 3.2 (Page 14)  Supplementary Table S1 and Supplementary Material 2 |
| Risk of bias in studies | 18 | Present assessments of risk of bias for each included study. | Section 3.8 (Page 17-18), Table 5, Supplementary Material S4 |
| Results of individual studies | 19 | For all outcomes, present, for each study: (a) summary statistics for each group (where appropriate) and (b) an effect estimate and its precision (e.g. confidence/credible interval), ideally using structured tables or plots. | Table 1,3 |
| Results of syntheses | 20a | For each synthesis, briefly summarise the characteristics and risk of bias among contributing studies. | Sections 3.3–3.6 (Pages 14-16) |
|  | 20b | Present results of all statistical syntheses conducted. If meta-analysis was done, present for each the summary estimate and its precision (e.g. confidence/credible interval) and measures of statistical heterogeneity. If comparing groups, describe the direction of the effect. | No meta-analysis conducted; narrative synthesis only |
|  | 20c | Present results of all investigations of possible causes of heterogeneity among study results. | Supplementary Figure S2 |
|  | 20d | Present results of all sensitivity analyses conducted to assess the robustness of the synthesized results. | Not applicable |
| Reporting biases | 21 | Present assessments of risk of bias due to missing results (arising from reporting biases) for each synthesis assessed. | Not assessed |
| Certainty of evidence | 22 | Present assessments of certainty (or confidence) in the body of evidence for each outcome assessed. | Not assessed |
| **DISCUSSION** | | |  |
| Discussion | 23a | Provide a general interpretation of the results in the context of other evidence. | Discussion section 4.1 – 4.10 (Pages 18-28) |
|  | 23b | Discuss any limitations of the evidence included in the review. | Discussion section 4.10 (Pages 27-28) |
|  | 23c | Discuss any limitations of the review processes used. | Discussion section 4.10 (Pages 27-28) |
|  | 23d | Discuss implications of the results for practice, policy, and future research. | Discussion section 4.10 (Pages 27-28) |
| **OTHER INFORMATION** | | |  |
| Registration and protocol | 24a | Provide registration information for the review, including register name and registration number, or state that the review was not registered. | The review protocol was not registered |
|  | 24b | Indicate where the review protocol can be accessed, or state that a protocol was not prepared. | The review protocol was not registered |
|  | 24c | Describe and explain any amendments to information provided at registration or in the protocol. | The review protocol was not registered |
| Support | 25 | Describe sources of financial or non-financial support for the review, and the role of the funders or sponsors in the review. | Funding Declaration Section |
| Competing interests | 26 | Declare any competing interests of review authors. | In conflict of Interest section |
| Availability of data, code and other materials | 27 | Report which of the following are publicly available and where they can be found: template data collection forms; data extracted from included studies; data used for all analyses; analytic code; any other materials used in the review. | Not Applicable. |

*From:*  Page MJ, McKenzie JE, Bossuyt PM, Boutron I, Hoffmann TC, Mulrow CD, et al. The PRISMA 2020 statement: an updated guideline for reporting systematic reviews. BMJ 2021;372:n71. doi: 10.1136/bmj.n71
